# Theoretical and Experimental Investigation of the Effect of Pump Laser Frequency Fluctuations on Signal-to-Noise Ratio of Brillouin Dynamic Grating Measurement with Coherent FMCW Reflectometry

**DOI:** 10.3390/s21082870

**Published:** 2021-04-19

**Authors:** Tatsuya Kikuchi, Ryohei Satoh, Iori Kurita, Kazumasa Takada

**Affiliations:** Department of Electronic Engineering, Faculty of Engineering, Gunma University, 1-5-1 Tenjin, Kiryu 376-8515, Gunma, Japan; t160d046@gunma-u.ac.jp (T.K.); t160d022@gunma-u.ac.jp (R.S.); t160d051@gunma-u.ac.jp (I.K.)

**Keywords:** fiber optics, fiber sensing, optical interference, nonlinear optics, four-wave mixing, Brillouin dynamic grating, optical reflectometry

## Abstract

Signal-dependent speckle-like noise has constituted a serious factor in Brillouin-grating based frequency-modulated continuous-wave (FMCW) reflectometry and it has been indispensable for improving the signal-to-noise ratio (S/N) of the Brillouin dynamic grating measurement to clarify the noise generation mechanism. In this paper we show theoretically and experimentally that the noise is generated by the frequency fluctuations of the pump light from a laser diode (LD). We could increase the S/N from 36 to 190 merely by driving the LD using a current source with reduced technical noise. On the basis of our experimental result, we derived the theoretical formula for S/N as a function of distance, which contained the second and fourth-order moments of the frequency fluctuations, by assuming that the pump light frequency was modulated by the technical noise. We calculated S/N along the 1.35 m long optical fiber numerically using the measured power spectral density of the frequency fluctuations, and the resulting distributions agreed with the measured values in the 10 to 190 range. Since higher performance levels are required if the pump light source is to maintain the S/N as the fiber length increases, we can use the formula to calculate the light source specifications including the spectral width and rms value of the frequency fluctuations to achieve a high S/N while testing a fiber of a given length.

## 1. Introduction

The Brillouin-enhanced four-wave mixing induced by counter-propagating pump lights and one probe light produces backward Stokes light at every location in an optical fiber under test [1], which is assumed to be the reflection of the probe light by the acoustic wave or Brillouin dynamic grating generated in the fiber by the two pump lights. Optical time-domain reflectometry (OTDR) has been used to detect the power of the Stokes light as a function of distance while changing the frequency difference between the pump lights to obtain the Brillouin spectrum distribution in the optical fiber [2,3,4,5,6]. A micrometer-scale spatial resolution is indispensable for diagnosing planar lightwave circuits (PLCs) [7] with the same four-wave mixing technique. Since the spatial resolution of the OTDR is determined by the temporal width of the employed optical pulses, a picosecond optical pulse should be launched into the optical fiber and detect return pulses without deformation by using a high-speed optical detector. Such an attempt to increase the spatial resolution often resulted in increased electrical noise due to the ultra-wide detection bandwidth over 10 GHz, and this degraded the signal-to-noise ratio (S/N). To our knowledge, therefore, there have been no reports on Brillouin dynamic grating measurement with a micrometer-scale spatial resolution using the OTDR method.

To overcome the S/N degradation, we have proposed Brillouin grating-based coherent frequency-modulated continuous-wave (FMCW) reflectometry [8], which is the frequency-domain counterpart of optical low coherence reflectometry (OLCR) [9] and it detects the power of the Stokes light by utilizing its interference with local oscillator (LO) light at a detection bandwidth of less than 1 MHz. We have succeeded in incorporating a fiber loop mechanism in conventional coherent FMCW reflectometry to generate the Stokes light [10,11,12]. Since the distance to the fiber segment where the Stokes light is generated is derived from the beat frequency, we do not have to use a mechanical stage such as that used in OLCR and thus we can extend the available distance range much further than can be achieved by the translation of the stage. Hereafter in this paper, the distribution of the Stokes light power along a fiber is referred to simply as a reflectogram from the fiber. Once we acquire reflectograms at various frequency differences, the Brillouin spectrum distribution along the optical fiber is obtained by changing the horizontal grid from equal distances to equal frequency intervals in the two-dimensional power data, which provides us with the strain distribution of the fiber.

Although we succeeded in demonstrating reflection measurement from a Brillouin dynamic grating with coherent FMCW reflectometry, we found signal-dependent multiplicative noise [13] to be the dominant noise, which meant that we could not improve the S/N merely by increasing the powers of the tunable laser output and pump lights and/or by narrowing the detection bandwidth. Hereafter in this paper, for convenience such multiplicative noise is referred to as speckle-like noise. Since the reflectograms that we derived from different measurements or made at different times had unavoidable speckle-like noise, we had no choice but to repetitively sweep the tunable laser source and add them to obtain a smoothed profile at every frequency difference. That is, we could obtain the desired Brillouin spectrum distribution and thus the strain distribution only after making a vast number of the repetitive sweeps over a long period of time. Therefore, to reduce the number of sweeps and the measurement time it was indispensable that we clarify the origin of the speckle-like noise and reduce it greatly.

This paper shows theoretically and experimentally that the speckle-like noise was generated by frequency fluctuations contained in the output light wave from the distributed feedback laser diode (DFB LD) used as the pump light source. Since the current from the employed current source injected into the LD had components that fluctuated with time, or technical noise, the resultant instantaneous frequency of the light output also fluctuated, and this generated the speckle-like noise in the reflectogram. First, we describe the experimental setup we employed for the coherent FMCW reflectometry system. Since silica-based PLC chips are usually between few millimeters and several tens of centimeters long, we adjusted the length of the optical fiber under test to be around 1 m. We constructed an unbalanced Mach-Zehnder interferometer (MZI) to obtain the power spectral density of the frequency fluctuations [14]. We employed two commercially available current sources from different manufacturers to drive the same LD, which had different power spectral density distributions. We used the density data to calculate the theoretical S/N and compared it with the experimental result.

Next, we theoretically derive the S/N dependence on the power spectral density of the frequency fluctuations on the condition that the absolute value of the complex amplitude of the acoustic wave or Brillouin dynamic grating was constant along the fiber. We calculated the second and fourth-order terms of the variance of the fluctuating Stokes light signal, which depended on the moments of the instantaneous frequency deviation of the pump light wave, and we derived the latter term as the correction of the former. Then, we compare the theoretical results with the experimental data obtained from a 1.35 m long optical fiber to confirm that the speckle-like noise was generated by the frequency fluctuations of the pump light waves. We showed that the second-order term provided us with the correct values for the S/N ranging from 10 to 190 when the coherence effect of the pump light wave was negligible. Then we measured the S/N distributions for 10 and 40 m long optical fibers. As the fiber length approached the coherence length of the pump light, the S/N values calculated with both the second and fourth-order terms no longer agreed with the measured value. Finally, we introduced the coherence function of the pump light wave into the second and fourth-order terms of the variance to explain the coherent effect and compared the calculated results with the data.

## 2. Experiment Setup and Basic Formulation

### 2.1. Experimental Setup for the Reflectometer

**Figure 1:**
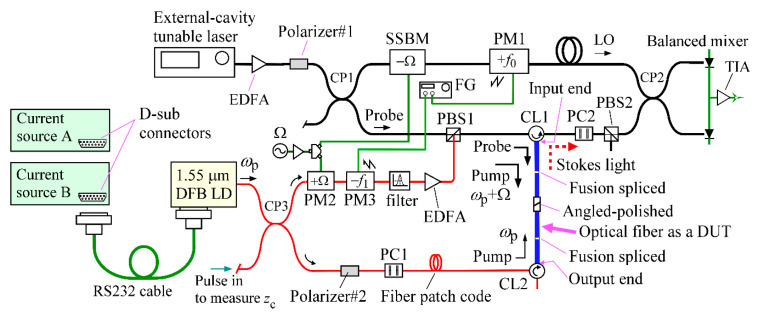
Schematic of Brillouin grating-based coherent FMCW reflectometry setup [15], which consisted of the conventional coherent FMCW reflectometry setup for detecting the reflection from a device under test (DUT) and an optical fiber loop for generating a Brillouin dynamic grating in the DUT. DFB LD: Distributed feedback laser diode, CL1 and CL2: Optical circulators, CP1, CP2 and CP3: 3-dB optical fiber couplers, EDFA: Erbium-doped fiber amplifier, PC1 and PC2: Polarization controllers, PBS1 and PBS2: Polarization beam splitters, SSBM: Single-sideband modulator (T.SBXH1.5-20PD-ADC, Sumitomo Osaka Cement), FG: 2-channel function generator, PM1, PM2 and PM3: LiNbO_3_ phase modulators (LN65S-FC, Thorlabs), LO: Local oscillator, TIA: Transimpedance amplifier, *ω*_p_: Pump light frequency, Ω: Up-conversion frequency of the pump light which is equal to the down-conversion frequency of the LO light, *f*_0_ = 150 kHz, *f*_1_ = 190 kHz. Picosecond pulses were launched into the fiber loop to locate the position at *z*_c_ where the optical path lengths of the counter-propagating pump light waves were equal.

The Brillouin grating-based coherent FMCW reflectometer comprised the conventional coherent FMCW reflectometry setup [10,11,12] for detecting the reflection from a device under test (DUT) and an optical fiber loop [2,3,4,5,6] for generating an acoustic wave or Brillouin dynamic grating in the DUT by counter-propagating pump lights, as shown in Figure 1. The conventional FMCW part was designed to detect the reflection whose optical frequency was down-converted by the Brillouin dynamic grating induced in the DUT. To achieve this detection, we incorporated a single-sideband modulator (SSBM) in the LO arm to down-convert the LO light. The power of the probe light launched into the DUT was 60 mW. The photocurrent output from the balanced mixer was converted to a voltage with a transimpedance amplifier (TIA) to obtain the beat signal waveform produced by interference between the Stokes light and the LO light. We drove the LiNbO_3_ phase modulators of PM1 and PM3 at *f*_0_ = 150 kHz and *f*_1_ = 190 kHz, respectively, with sawtooth voltage waveforms supplied by a two-channel function generator so that the carrier frequency of the beat signal waveform was *f*_1_ − *f*_0_ = 40 kHz.

Although not shown in Figure 1, some of the tunable laser output was supplied to an auxiliary unbalanced fiber-optic MZI to change the grid of the interference fringe produced during the frequency sweep from equal time to equal frequency increments. The time delay between the two arms of the MZI was adjusted to 58.449 ns. We sampled the beat signal waveform from the balanced mixer twice when the reference beat signal from the MZI traveled one cycle. The DUT, which is shown in blue in Figure 1, consisted of four pieces of single-mode fiber. Two of them were fiber pigtails attached to optical circulators CL1 and CL2 and the rest were fiber patch cords with angled physical contact (APC) connectors. We spliced each pigtail to either patch cord using fusion splicing and mated the connectors together with an SC/APC adapter. The total length of the DUT was *L* = 1.35 m and the bases of the fiber pigtails at the CL1 and CL2 were denoted as the input and output ends of the DUT, respectively. We used a configuration consisting of a pair of polarization controllers (PC1 and PC2) and a polarization beam splitter (PBS2) to block the pump light from entering the balanced mixer [15].

In the optical fiber loop, we used a DFB LD operating at 1550 nm as the pump light source, which was driven by two commercially available current sources that we refer to as current sources A and B. We used current source A for the reflection measurements described in our previous papers [8,15], whereas we prepared current source B specifically for use in this experiment. The LD was packaged in an aluminum case where a D-sub connector was attached to supply injection current to the LD and control current to a thermoelectric cooler installed in the LD module. We selected either current source as the LD driver by connecting the D-sub connector at the LD case with that of the current source via RS232 cable.

The LD output was divided into two with an optical fiber coupler (CP3) for use as two counter-propagating pump lights. We describe the fixed frequency of the laser output as *ω*_p_ in the figure. To up-convert the frequency of the pump light by the same frequency Ω as the down-conversion frequency of the probe light, we applied phase modulation at Ω to the pump light with a LiNbO_3_ phase modulator (PM2) and extracted the up-converted light component with an optical narrow-band filter. The resultant pump light at *ω*_p_ + Ω was amplified with an erbium-doped fiber amplifier (EDFA), combined with the probe light at a polarization beam splitter (PBS1), and launched into the DUT after passing through the optical circulator CL1, where the launched power was 120 mW. The other laser output was launched into the DUT from the opposite direction for use as a pump light at *ω*_p_ after passing through the optical circulator CL2.

**Figure 2:**
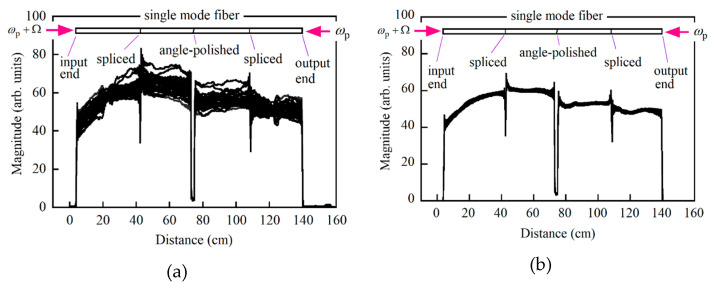
Overwritten reflectograms from a 1.35 m long optical fiber that were obtained by 30 frequency sweeps of a tunable laser when the pump LD was driven with (**a**) current source A and (**b**) current source B. The up-conversion frequency was 10.861 GHz.

We drove the pump LD with the current source A and acquired 30 reflectograms from the 1.35 m long fiber by repeatedly sweeping the tunable laser, as shown in Figure 2a, where the up-conversion frequency was 10.861 GHz. The optical fiber had two spliced points and one mated point where the Brillouin frequency shifts had been moved downward so that the Stokes signals at those points decreased rapidly. With current source A, the signal changed greatly with every sweep, and we had to obtain a smooth profile by adding individual reflectograms. We then drove the same LD with another current source, B, while maintaining the same measurement condition and found that the signal variations were greatly reduced, as shown in Figure 2b. By comparing these results, we considered that the noise contained in the current injected into the LD produced the fluctuations in the optical frequency of the pump light, which resulted in the signal variations. We placed the optical fiber under test straight on an optical bench in a normal laboratory environment, but the Stokes signal changed gradually along the fiber. This was because the optical fiber was not a polarization-maintaining fiber and the states of polarization (SOPs) of the pump lights and the probe light changed along the fiber, resulting in changes in both the amplitude and SOP of the Stokes light.

### 2.2. Basic Formulation Using Instantaneous Frequency Shift

**Figure 3:**
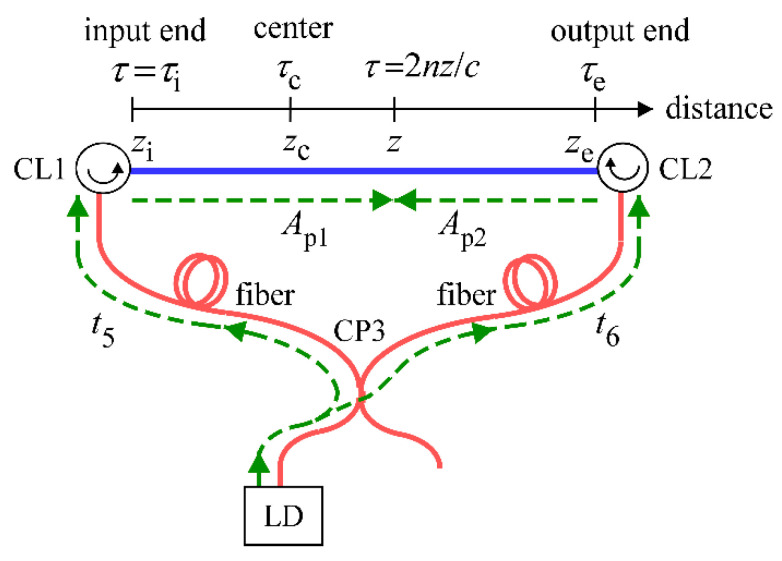
Schematic of the propagation (highlighted in green) of counter-propagating pump light waves from the LD in the fiber loop. *t*_5_ and *t*_6_ are the propagation times of the pump light waves from the LD to optical circulators CL1 and CL2, respectively. *z* is a coordinate of the distance along the fiber under test highlighted in blue and the origin of the distance is positioned at the point where the produced reflection has the same optical path length as the LO light at the balanced mixer. The input and output ends of the fiber were assumed to be located at *z*_i_ and *z*_e_, respectively. *τ* is the round-trip time from the origin to any position at *z* as defined by *τ* = 2*nz*/*c*, where *n* is the refractive index of the fiber and *c* is the velocity of light in a vacuum. Similarly, *τ*_i_ and *τ*_e_ are defined as *τ*_i_ = 2*nz*_i_/*c* and *τ*_e_ = 2*nz*_e_/*c*. *A*_p1_ and *A*_p2_ are the complex amplitudes of the electric fields of the pump light waves propagating in the clockwise and counterclockwise directions, respectively. The center of pumping is defined by the position where the optical path lengths of the two pump light waves are equal in the optical fiber loop. CL1 and CL2: Optical circulators, CP3: Fiber coupler, LD: Laser diode.

In Figure 3, the light propagation of the two pump light waves in the fiber loop is shown with green dotted lines, and the optical fiber as the DUT is shown in blue. The origin of the distance *z* is chosen at the point where the optical path lengths of the LO light and the reflection from the point are equal. We assume that the fiber distributed from the input end at *z* = *z*_i_ to the output end at *z* = *z*_e_ so that the total length was *z*_e_ − *z*_i_ = *L* = 1.35 m. The acoustic field at time *t* and distance *z* excited by material density fluctuations in the DUT is described as *ρ*(*z*,*t*) = *ρ*_0_ + {*R*(z)exp[*i*(*qz* − Ω*t*)] + c.c.} [1], where *ρ*_0_ is mean density, *q* is the wavenumber of the acoustic field, Ω is the frequency difference between the counter-propagating pump light waves, *i* is pure imaginary and c.c. denotes the complex conjugate.

Under a steady-state condition, the amplitude of the acoustic wave is denoted as:

(1) $$R(z)=\frac{\varepsilon_{0}\gamma_{e}q^{2}A_{p1}A_{P2}^{*}}{\Omega_{B}^{2}-\Omega^{2}-i\Omega \Gamma_{B}},$$

where *ε*_0_ is the vacuum permittivity, *γ_e_* is the electrostrictive constant of the DUT material, Ω_B_ is the center frequency of the Brillouin spectrum as a function of *z*, which is to be measured with the reflectometer, and Γ_B_ is the Brillouin linewidth. When the amplitude of the electric field of the LD output at *t* is denoted as *A*(*t*), the amplitudes of the counter-propagating pump light waves at (*t*,*z*) are represented by *A*_p1_ = *c*_1_*A*(*t* − *t*_5_ − *nz*/*c*) and *A*_p2_ = *c*_2_*A*(*t* − *t*_6_ − *n*(*L* − *z*)/*c*), where *c*_1_ and *c*_2_ are constants, and *t*_5_ and *t*_6_ are the times required for these light waves to propagate from the LD to the optical circulators CL1 and CL2, respectively. It should be noted that we had already introduced parameters *t*_1_ *t*_2_, *t*_3_ and *t*_4_ as the propagation times in the reflectometer setup in our previous paper [8] and thus in this paper we introduce new parameters *t*_5_ and *t*_6_ to avoid confusion. The phases of both waves always agree with each other at a point *z* = *z*_c_ = *L*/2 + *c*(*t*_6_ − *t*_5_)/2*n*, which is hereafter referred to as the center of pumping. Here we introduce *τ* and *τ*_c_ as the round-trip times from the origin to a point at *z* and to the center of pumping at *z*_c_, which are given by *τ* = 2*nz*/*c* and *τ*_c_ = 2*nz*_c_/*c*, respectively. By shifting the origin of time *t* by *t*_5_, the amplitudes of the counter-propagating pump light waves at (*t*, *z*) are denoted as *A*_p1_ = *c*_1_*A*(*t* − *τ*/2) and *A*_p2_ = *c*_2_*A*(*t* − *τ*_c_ + *τ*/2).

If the phase of the output light wave from the LD is modulated by the technical noise contained in the injection current, we can denote its amplitude as *A*(*t*) = *A*_0_exp(−*iϕ*(*t*)) with a constant *A*_0_, and thus *A*_p1_*A*_p2_* in Equation (1) is written as *A*_p1_*A*_p2_ * = *c*_1_*c*_2_ *A(*t*−*τ*/2)*A* *(*t* − *τ*_c_ + *τ*/2) = *c*_1_*c*_2_ *|*A*_0_|^2^exp{− *i*[*ϕ*(*t* − *τ*/2)*-ϕ*(*t* − *τ*_c_ + *τ*/2)]}, where the phase difference is expanded to *ϕ*(*t* − *τ*/2) − *ϕ*(*t* − *τ*_c_ + *τ*/2)= *ϕ*′(*t*)(*τ*_c_ − *τ*) + *ϕ*′′(*t*)(*τ* − *τ*_c_)*τ*_c_/2 + … That is, the absolute value of the complex amplitude of the acoustic wave is unchanged, but its temporal phase is modulated by the technical noise. When we make an approximation where *ϕ*(*t* − *τ*/2) − *ϕ*(*t* − *τ*_c_ + *τ*/2) ≈ *ϕ*′(*t*)(*τ*_c_ − *τ*), the approximation error is of the order of the product of the root-mean-square value of *ϕ*′′(*t*) and the maximum value of |(*τ* − *τ*_c_)*τ*_c_|/2.

Since the phase induced by the current noise is generally represented by *ϕ*(*t*) = ∑*ϕ_k_*cos2*πf_k_t*, we have *ϕ*′(*t*) = −2*π*∑*δν_k_*sin2*πf_k_t* and thus *ϕ*′′(*t*) = −(2*π*)^2^∑*δν_k_f_k_*cos2*πf_k_t*, where *δν_k_* is the maximum frequency deviation at *f_k_*. Since *ϕ*′(*t*)/2*π* is the instantaneous frequency deviation of the pump light wave from the center frequency, its mean square value, which is denoted as *δν*_rms_^2^, is calculated to be *δν*_rms_^2^ = ∑*δν_k_*^2^/2. Similarly, we have <(*ϕ*′′(*t*))^2^ > = (2*π*)^4^∑*δν_k_*^2^*f_k_*^2^/2, which should be less than (2*π*)^4^*f*_u_^2^∑*δν_k_*^2^/2 = (2*π*)^4^*f*_u_^2^*δν*_rms_^2^, where *f*_u_ is the upper limit of the frequency contributing to the speckle-like noise. The result shows that we can estimate the root mean square value of *ϕ*′′(*t*) as √ < (*ϕ*′′(*t*))^2^> ≈ (2*π*)^2^*f*_u_*δν*_rms_. On the other hand, the factor |(*τ*−*τ*_c_)*τ*_c_|/2 is of the order of *τ*_e_^2^/8 because *τ*_e_ ≈ 0 and thus *τ*_c_ ≈ *τ*_e_/2 and 0 < *τ* < *τ*_e_. Therefore, we find that the approximation error is of the order of {√ < (*ϕ*′′(*t*))^2^ >}*τ*_e_^2^/8 = *π*^2^*f*_u_*δν*_rms_*τ*_e_^2^/2. In our experiment we adopt *τ*_e_ = 13.5 ns, *f*_u_ ≈ 1 kHz, and *δν*_rms_ ≈ 2 MHz for the current source A, as will be described in Section 4, and thus the approximation error is estimated to be 1.8 × 10^−6^ rad, meaning that the accuracy of the approximation *ϕ*(*t* − *τ*/2) − *ϕ*(*t* − *τ*_c_ + *τ*/2) ≈ *ϕ*′(*t*)(*τ*_c_ − *τ*) is very high and the error is negligibly small.

Since *ϕ*′(*t*) represents the instantaneous angular frequency deviation from *ω*_p_, we denote it as Δ(*t*) so that we have a good approximation of *ϕ*(*t* − *τ*/2) − *ϕ*(*t* − *τ*_c_ + *τ*/2) ≈ Δ(*t*)(*τ*_c_ − *τ*). We have already derived the expression for the power of the Stokes light as a function of *τ* [8]. With this approximation, the power is obtained by calculating the absolute square of the Fourier inverse transform of the following analytic signal *I*(*t*) with respect to *ω*(*t*):

(2) $$I(t)=\int_{-\infty }^{+\infty }r(\tau_{1})e^{-i\Delta (t)(\tau_{c}-\tau_{1})}e^{-i\left(\omega_{1}+\omega (t)-\omega_{p}\right)\tau_{1}}\;d\tau_{1},$$

where the proportionality coefficient of the signal is included in *r*(*τ*) for simplicity so that *r*(*τ*) is the complex function of *τ* which is independent of the phase modulation due to technical noise. *ω*_1_ and *ω*_1_ + *ω*(*t*) are the start frequency and the instantaneous frequency at time *t* of the tunable laser output, respectively. The phase of the analytic signal changed with time due to Δ(*t*), resulted in the fluctuations of the Stokes light signal.

### 2.3. Experimental Setup for Measuring Power Spectral Density

**Figure 4:**
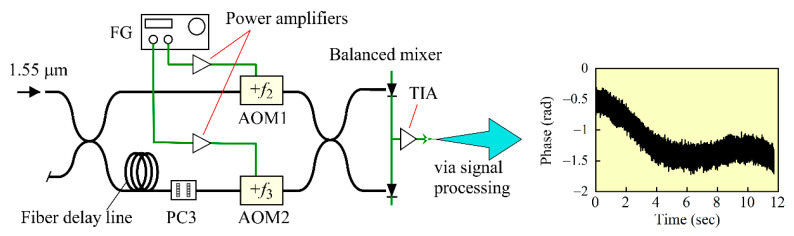
Schematic of an unbalanced Mach-Zehnder interferometer (MZI) used to measure frequency fluctuations of the light that was incident from the input port of the MZI. AOM1 and AOM2: Acousto-optic frequency shifters, FG: Function generator, PC3: Polarization controller, TIA: Transimpedance amplifier. *f*_2_ and *f*_3_ are up-conversion frequencies by the AMO1 and AOM2, respectively. PC3 was adjusted for the amplitude of the beat signal to reach its maximum value. The inset shows a typical phase change waveform, which was retrieved from the acquired beat signal waveform.

We define the signal-to-noise ratio or S/N of the reflection measurement by the ratio of the mean level of the fluctuating powers of the Stokes light signal to their standard deviation, which is defined by the square root of the variance. Since the variance depends on the power spectral density of the angular frequency fluctuations Δ(*t*), we constructed an unbalanced fiber-optic MZI as shown in Figure 4 to measure the power spectral density [14,16,17,18,19]. Since we did not have two more LiNbO_3_ phase modulators for frequency up-conversion, we installed acousto-optic frequency modulators (AOM1 and AOM2) in the two arms of the interferometer and drove them so that the frequency difference between the up-conversion frequencies in both arms became the same at 40 kHz. By connecting a fiber patch cord as a fiber delay line to one arm of the interferometer, we adjusted the time delay between the two arms to *τ*_MZI_ *=* 8.912 ns.

We launched the output from the DFB LD into the MZI and sampled the beat signal from the balanced mixer at a rate of 256 k samples/s for 11.7 s. We calculated the Fourier transform of the acquired waveform from which we removed the carrier frequency and calculated the Fourier inverse transform of the result to obtain the analytic signal. Then we unwrapped the phase term of the analytic signal and obtained the temporal change of the phase, as shown by the inset in Figure 4. The overall profile of the retrieved phase change tended to decay gradually and then remained constant mainly due to environmental perturbations applied to the MZI during the measurement. Considering that the delay time *τ*_MZI_ was shorter than *τ*_e_, the phase of the analytic signal is given by Δ(*t*)*τ*_MZI_, and so we could obtain the actual change of Δ(*t*) by dividing the measured phase change by the delay *τ*_MZI_. It is noted that the temporal change of the light frequency, *δν*(*t*), is given by Δ(*t*)/2*π*.

**Figure 5:**
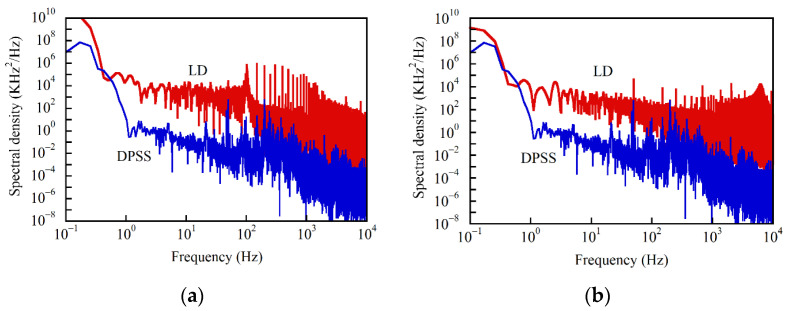
Power spectral density (shown in red) of the frequency fluctuations of the LD output when it was driven with (**a**) current source A and (**b**) current source B. The power spectral density obtained from a diode-pumped solid-state (DPSS) laser is also plotted in blue in each figure.

We calculated the power spectral density *H*(*f*) of the temporal frequency change by applying Fourier transformation to *δν*(*t*) which was given by Δ(*t*)/2π, as shown in Figure 5a,b, where the LD was driven with current sources A and B, respectively, and the sampling interval was 0.0853 Hz. For comparison, in each figure we plotted the spectral density, which we obtained from a low noise continuous wave diode-pumped solid state (DPSS) laser operating at 1.34 μm. Each spectral density from the LD contained a vast number of peaks that were superimposed on the gradually decaying background component. Since all the components ranging from 1 Hz to 10 kHz were larger than the background noise level of the spectral density of the DPSS laser, we concluded that they all originated from the frequency fluctuations of the LD itself, not from noise in the detection system.

Over a 100 Hz to 1 kHz frequency range, we observed more intense peaks in the spectral density (a) than in (b), which we considered to be the main origin of the larger fluctuations in the reflectograms in (a) than in (b) of Figure 2. As observed in the inset in Figure 4, the phase changed gradually for 11.7 s mainly due to external perturbations applied to the interferometer, and thus we concluded that the spectral components ranging from 0.1 to 1 Hz in both Figure 5a,b arose not from the actual frequency fluctuations, but from the environmental perturbations.

## 3. Calculation

In this section we derive the mean level and standard deviation of the fluctuating power of the Stokes light signal to obtain the S/N of the reflection measurement. We assume that the frequency of the tunable laser output sweeps from the start frequency *ω*_1_ sufficiently linearly with time to meet *ω*(*t*) = *βt* with a constant *β*. To simplify the calculation of the Fourier inverse transform of Equation (2) with respect to *ω*(*t*), we introduce a new variable *ῶ*, which is defined by *ω*_1_ + *ω*(*t*) − *ω*_p_ = *ῶ*, so that *t* is a function of *ῶ* represented by *t* =(*ῶ* + *ω*_p_ − *ω*_1_)/*β*. By expanding the exponential function in the integrand of *I*(*t*) in power series up to the fourth order, *I*(*t*) is approximated as:

(3) $$I\left(t\right)=\sum_{k=0}^{4}\frac{{\left(-i\right)}^{k}}{k!}\int_{-\infty }^{+\infty }r(\tau_{1})\Delta^{k}(t){\left(\tau_{c}-\tau_{1}\right)}^{k}e^{-i\varpi \tau_{1}}\;d\tau_{1}.$$

The Fourier inverse transform of *I*(*t*) with respect to *ῶ* is then written as:

(4) $$\frac{1}{2\pi }\int_{-\infty }^{+\infty }I\left(t_{1}\right)e^{i\varpi_{1}\tau }d\varpi_{1}=\sum_{k=0}^{4}X^{\left(k\right)},$$

where *X*^(0)^ = *r*(*τ*) and:

(5) $$X^{\left(k\right)}=\frac{{\left(-i\right)}^{k}}{2\pi k!}\int_{-\infty }^{+\infty }\int_{-\infty }^{+\infty }r(\tau_{1})\Delta^{k}(t_{1}){\left(\tau_{c}-\tau_{1}\right)}^{k}e^{i\varpi_{1}\left(\tau -\tau_{1}\right)}\;d\tau_{1}d\varpi_{1}\;(k=1∼4),$$

and where *t*_1_ = (*ῶ*_1_ + *ω*_p_ − *ω*_1_)/*β*. Hereafter we abbreviate *r*(*τ*) as *r* when there is no confusion.

*X*^(0)^ = *r*(*τ*) is independent of the random process, whereas *X*^(*k*)^ (*k* ≥ 1) is a random variable that contains the *k*th power of Δ(*t*) in the integrand. From Equation (4) the absolute square is given by:

(6) $$Z={\left|\sum_{k=0}^{4}X^{\left(k\right)}\right|}^{2}=\sum_{k=0}^{4}\sum_{j=0}^{4}X^{\left(k\right)}X^{(j)*},$$

which provides the mean value of *Z* as:

(7) $$\langle Z\rangle ={\left|r\right|}^{2}+\left[r*\left(\langle X^{\left(2\right)}\rangle +\langle X^{\left(4\right)}\rangle \right)+c.c.\right]+\langle {\left|X^{\left(1\right)}\right|}^{2}\rangle +\langle {\left|X^{\left(2\right)}\right|}^{2}\rangle .$$

when we decompose *Z* into <*Z*> +*δZ*, the variance *σ*^2^ of *Z* is <(*δZ*)^2^ >, which is represented by:

(8) $$\sigma^{2}=\langle {\left(Y^{\left(1\right)}+Y_{1}^{\left(2\right)}+Y_{2}^{\left(2\right)}+Y^{\left(3\right)}+Y_{1}^{\left(4\right)}+Y_{2}^{\left(4\right)}\right)}^{2}\rangle .$$

The calculations for deriving Equations (7) and (8) are detailed in Appendix A, where the summands in Equation (8) are defined by Equations (A5) to (A10). The superscript *k* in *Y*^(*k*)^ denotes that *Y*^(*k*)^ contains the *k*th power of Δ(*t*) in the integral.

In accordance with the Wiener–Khinchin theorem [20,21], we introduce the power spectral density *G*(*χ*) of Δ(*t*), which is defined by:

(9) $$\langle \Delta (t_{1})\Delta (t_{2})\rangle =\int_{-\infty }^{+\infty }G(\chi )e^{-i\chi \left(t_{1}-t_{2}\right)}\;d\chi ,$$

where *G*(*χ*) should be a real-valued and even function of the angular frequency *χ*. By letting Δ(*t*) = 2*πδν*(*t*) and *χ* = 2π*f,* Equation (9) is changed to <*δν*(*t*_1_)*δν*(*t*_2_) > = (1/2π)∫_−∞_^+∞^ *G*(2*πf*)exp[−2π*if*(*t*_1_ − *t*_2_)]*df*, where *δν*(*t*) is the instantaneous frequency deviation of the pump light and *f* is frequency, and thus we find the relational expression of *G*(*χ*) = 2*πH*(*χ*/2*π*) between *G*(*χ*) and the power spectral density *H*(*f*) of *δν*(*t*). It is noted that each plot in Figure 5 shows *H*(*f*) measured when we drove the LD with either current source A or current source B. The mean square value of *δν*(*t*), which we denote as *δν*_rms_^2^, is represented by the infinite integral of *H*(*f*) with respect to *f*. Actually, however, the detection bandwidth for the Stokes light signal was limited to *f*_u_ = *βτ*_e_/2*π* so that *δν*_rms_^2^ must be evaluated by taking the finite integral over (−*f*_u_, *f*_u_), which is changed to the integral over (0, *f*_u_) as:

(10) $$\delta \nu_{\mathrm{rms}}^{2}=2\int_{0}^{f_{u}}H(f)df,$$

because *H*(*f*) is an even function of *f*. Equation (10) means that *δν*_rms_ depends on the length of the fiber under test and is related to Δ_rms_, which is the root mean square value of Δ(*t*) via Δ_rms_ = 2*πδν*_rms_.

To express <*Z*> using *G*(*χ*), we must calculate the following double integral at *k* = 2 and 4:

(11) $$\langle X^{\left(k\right)}\rangle =\frac{{\left(-i\right)}^{k}}{2\pi k!}\langle \Delta^{k}(t)\rangle \int_{-\infty }^{+\infty }\int_{-\infty }^{+\infty }r(\tau_{1}){\left(\tau_{c}-\tau_{1}\right)}^{k}e^{i\varpi_{1}\left(\tau -\tau_{1}\right)}\;d\tau_{1}d\varpi_{1}.$$

By assuming that Δ(*t*) is a zero-mean Gaussian random variable, we obtain <Δ^4^(*t*)> = 3Δ_rms_^4^ with Δ_rms_^2^ = <Δ^2^(*t*)> according to the moment theorem [21,22,23]. In the double integral in Equation (11), first we take the iterated integral with respect to *ῶ*_1_ to obtain 2*πδ*(*τ* − *τ*_1_), where *δ*(*τ*) is the delta function. Then we integrate the resultant integrand including the delta function with respect to *τ*_1_ to obtain <*X*^(2)^ > = − *r*(*τ*)Δ_rms_^2^(*τ*_c_ − *τ*)^2^/2 and <*X*^(4)^> = *r*(*τ*)Δ_rms_^4^(*τ*_c_ − *τ*)^4^/8. When we neglect the last two terms <|*X*^(1)^|^2^> + <|*X*^(2)^|^2^> in Equation (7), we have <*Z*> ≈ |*r*(*τ*)|^2^[1 − Δ_rms_^2^(*τ*_c_ − *τ*)^2^ + Δ_rms_^4^(*τ*_c_ − *τ*)^4^/4], which is approximated by the Gaussian function as:

(12) $$\langle Z\rangle \approx {\left|r(\tau )\right|}^{2}e^{-{\left[\Delta_{\mathrm{rms}}\left(\tau_{c}-\tau \right)\right]}^{2}}.$$

It is noted that the actual third terms of the power series expansion of the Gaussian function should be Δ_rms_^4^(*τ*_c_ − *τ*)^4^/2.

From Equation (8), *σ*^2^ is expanded to <*Y*^(1)2^> + 2 < *Y*^(1)^(*Y*_1_^(2)^ + *Y*_2_^(2)^)> + 2 < *Y*^(1)^*Y*^(3)^> + <(*Y*_1_^(2)^ + *Y*_2_^(2)^)^2^> + higher-order terms, where the second term vanishes because the integrand of it contains the third-order moment of Δ(*t*). Then the variance is approximated by adding the second-order term *σ*^(2)^ and fourth-order term *σ*^(4)^ as:

(13) $$\sigma^{2}=\sigma^{\left(2\right)}+\sigma^{\left(4\right)},$$

where:

(14) $$\sigma^{\left(2\right)}=\langle Y^{\left(1\right)}{}^{2}\rangle ,$$

(15) $$\sigma^{\left(4\right)}=2\langle Y^{\left(1\right)}Y^{\left(3\right)}\rangle +\langle {\left(Y_{1}^{\left(2\right)}+Y_{2}^{\left(2\right)}\right)}^{2}\rangle .$$

*σ*^(4)^ is considered to be a correction term when we estimate the variance from Equation (14).

We calculated *σ*^(2)^ and *σ*^(4)^ by assuming that Δ(*t*) obeys a Gaussian random process as described in Appendix B and Appendix C, respectively. Because |*σ*^(4)^| < < *σ*^(2)^ as long as we measure the 1.35 m long optical fiber as will be shown in Section 4, here we describe the result of *σ*^(2)^ only:

(16) $$\begin{array}{l} \sigma^{\left(2\right)}=2{\left|r(\tau )\right|}^{2}{\int_{-\infty }^{+\infty }{\left|r\left(\tau -\frac{\chi }{\beta }\right)\right|}^{2}\left(\tau_{c}-\tau +\frac{\chi }{\beta }\right)}^{2}G(\chi )\;d\chi \\ \;\;\;\;\;\;\;+\left\{-r^{*}{}^{2}(\tau )\int_{-\infty }^{+\infty }r\left(\tau -\frac{\chi }{\beta }\right)r\left(\tau +\frac{\chi }{\beta }\right)\left[{\left(\tau_{c}-\tau \right)}^{2}-{\left(\frac{\chi }{\beta }\right)}^{2}\right]G(\chi )\;d\chi +c.c.\right\}. \end{array}$$

In general, the variance depends on the distribution *r*(*τ*), which changes along the fiber in an actual field application, and this means that it would be rather difficult to calculate the integrals in Equation (16). Since our aim in this paper is to clarify the origin of the speckle-like noise that we observed in the reflection measurement, we assume that the fiber under test is uniform and tension-free throughout the fiber and thus *r*(*τ*) is a constant function of *τ*, or *r*(*τ*) = *r*_0_ in the range *τ*_i_ ≤ *τ*≤ *τ*_e_ and *r*(*τ*) = 0 elsewhere. The integrands of the first and second terms in Equation (16) contain the functions of *r*(*τ* − *χ*/*β*) and *r*(*τ* − *χ*/*β*)*r*(*τ* + *χ*/*β*), which are finite only when the variable *χ* satisfies the conditions of *τ*_i_ ≤ *τ*−*χ*/*β* ≤ *τ*_e_, and *τ*_i_ ≤ *τ* ± *χ*/*β* ≤ *τ*_e_, respectively. Then <*Z*> and √*σ*^(2)^ are calculated to be proportional to |*r*_0_|^2^, which is dropped when taking the ratio of <*Z*> to √*σ*^(2)^ to obtain S/N. For this reason, we redefined *σ*^(2)^ as the one which is obtained by letting |*r*_0_| = 1 in the original expression. The explicit forms of *σ*^(2)^ for *τ*_i_ < *τ* < (*τ*_i_ + *τ*_e_)/2 and (*τ*_i_ + *τ*_e_)/2 < *τ* < *τ*_e_ are given by Equations (A42) and (A43), respectively, in Appendix D. The S/N including the fourth-order term *σ*^(4)^ is calculated in Appendix E.

By introducing the variable *u* and parameters *u*_c_ and *u*_i_, which are defined as *u* = *τ*/*τ*_e_, *u*_c_ = *τ*_c_/*τ*_e_ and *u*_i_ = *τ*_i_/*τ*_e_, the ranges of *τ* for *τ*_i_ < *τ* < (*τ*_i_ + *τ*_e_)/2 and (*τ*_i_ + *τ*_e_)/2 < *τ* < *τ*_e_ are changed to those of *u* for *u*_i_ < *u* < (*u*_i_ + 1)/2, and (*u*_i_ + 1)/2 < *u* < 1, respectively. Then the mean signal level described by Equation (12) is rewritten as:

(17) $$\langle Z\rangle \approx e^{-{\left[\Delta_{\mathrm{rms}}\tau_{e}\left(u_{c}-u\right)\right]}^{2}}.$$

By letting |*r*(*τ*)| = 1 and thus S/N for *u*_i_ < *u* < (*u*_i_ + 1)/2 is described with the variance as:

(18) $$\frac{S}{N}=\frac{e^{-{\left[\Delta_{\mathrm{rms}}\tau_{e}\left(u_{c}-u\right)\right]}^{2}}}{\sqrt{\sigma^{\left(2\right)}}},$$

where:

(19) $$\sigma^{\left(2\right)}=2\tau_{e}^{3}\beta \left[\int_{u-u_{i}}^{1-u}{\left(u_{c}-u-\eta \right)}^{2}G(\beta \tau_{e}\eta )\;d\eta +4\int_{0}^{u-u_{i}}\eta^{2}G(\beta \tau_{e}\eta )\;d\eta \right],$$

and where *η* = *χ*/(*βτ*_e_) = *f*/*f*_u_, *f*_u_ = *βτ*_e_/2*π* and *G*(*βτ*_e_*η*) = 2*πH*(*f)*. To obtain the expression for (*u*_i_ + 1)/2 < *u* < 1, in Equation (19) we should change the interval of the first integral to (1 − *u*, *u* − *u*_i_) and that of the second integral to (0, 1 − *u*), and change the sign of the variable *η* in the integrand of the first term.

Since the power spectral density *H*(*f*) of the frequency fluctuations had a vast number of peaks due to the technical noise from the current sources, as shown in Figure 5, to evaluate Δ_rms_ and *σ*^(2)^ we should calculate Equation (19) numerically using the measured power spectral density. In Appendix F, we calculated *σ*^(2)^ analytically on the assumption that the noise was concentrated on a narrow region expressed by a Gaussian function *G*(*χ*) = *G*_0_exp[− (*χ*/*δχ_e_*)^2^] using *δχ_e_* for the spectral half width at 1/*e* maximum and that the fiber under test was sufficiently long for the fiber input end and the center of pumping to be *τ*_i_ ≈ 0 and *τ*_c_ ≈ *τ*_e_/2, respectively, so that we can let *u*_i_ = 0 and *u*_c_ = 1/2. S/N at an arbitrary value of *α* = *βτ*_e_/*δχ_e_* is obtained by substituting *σ*^(2)^ represented by Equation (A78) into Equation (18) for 0 < *u* < 1/2, or by substituting that represented by Equation (A80) into Equation (18) for 1/2 < *u* < 1.

Although the analytical solution to *σ*^(2)^ had a complicated form, we could approximate it as represented by Equation (A83) and it is valid that:

(20) $${\left(\frac{S}{N}\right)}_{\infty }=\sqrt{\frac{\ln2}{2}}\frac{\gamma e^{-{\left[\Delta_{\mathrm{rms}}\tau_{e}\left(u_{c}-u\right)\right]}^{2}}}{2\pi f_{h}\delta \nu_{\mathrm{rms}}}$$

at *α* ≥ *α*_min_ = 8 within a calculation error of around 20%, as will be shown numerically in Section 4. In Equation (20), *f*_h_ and *γ*, respectively, are the spectral half width at a half maximum of the Gaussian spectrum defined by *f*_h_ = *δχ*_e_(√ln2)/2*π*, and the sweep rate of the tunable laser output defined by *γ* = *β*/2*π*.

*α* is denoted as (√ln2)*γτ*_e_/*f*_h_, which should be less than *α*_min_ so that the approximation of Equation (20) holds. To meet the condition, the range allowed for *f*_h_ should be:

(21) $$f_{h}\leq \frac{\sqrt{\ln2}}{\alpha_{\min}}\gamma \tau_{e}.\;$$

Because 0 < *u* < 1, the numerator in Equation (20) takes *γ* at *u* = *u*_c_ = 1/2 and decreases to *γ*exp[− (Δ_rms_*τ*_e_/2)^2^] at both ends, which is written as *γ*exp[− (*πδν*_rms_*τ*_e_)^2^] using the relational expression Δ_rms_ = 2*πδν*_rms_. Thus, we impose the second condition:

(22) $$\delta \nu_{\mathrm{rms}}\leq \frac{1}{2\pi \tau_{e}}\;$$

to regard the numerator as the constant *γ* so that the mean signal level is kept constant throughout the fiber. Then, (*S*/*N*)_∞_ is simplified to √(ln2/2)*γ*/(2*πf*_h_*δν*_rms_), which should be equal to or greater than the target value of (*S*/*N*)_tgt_, and so we found that *δν*_rms_ and *f*_h_ must satisfy the third condition:

(23) $$\delta \nu_{\mathrm{rms}}\leq \frac{1}{2\pi }\sqrt{\frac{\ln2}{2}}\frac{\gamma }{{\left(\frac{S}{N}\right)}_{\mathrm{tgt}}}\cdot \frac{1}{f_{h}}$$

at given values of *γ* and (*S*/*N*)_tgt_.

## 4. Experimental Results

We fixed the up-conversion frequency of the pump light wave at 10.861 GHz, which was the center frequency of the Brillouin spectrum of the 1.35 m long optical fiber under test. The parameters *u*_i_ and *u*_c_ were originally defined by *u*_i_ = *τ*_i_/*τ*_e_ and *u*_c_ = *τ*_c_/*τ*_e_ and were rewritten as *u*_i_ = *z*_i_/*z*_e_ and *u*_c_ = *z*_c_/*z*_e_ because of the linearity between the propagation time and distance, or *τ*_i_ = 2*nz*_i_/*c*, *τ*_c_ = 2*nz*_c_/*c* and *τ*_e_ = 2*nz*_e_/*c*. We calculated the mean reflectogram from the 30 reflectograms shown in Figure 2a and measured the distances to the points where the Stokes signal raised and fell to give *z*_i_ = 4.11 cm and *z*_e_ = 139.8 cm. With these values, we obtained *u*_i_ = 0.0294. To locate the center of pumping, we launched a picosecond optical pulse from another input port of fiber coupler CP3 into the optical fiber loop and measured the propagation times during which the counter-propagating optical pulses reached the two angled-polished end faces of the optical fiber under test. Since the center of pumping was defined by the position where the optical path lengths of the counter-propagating pump lights were equal in the fiber loop, the distance *z*_c_ to the center of pumping could be determined by the difference between the propagation times of the pulses. Since the distance increased by connecting a longer optical fiber delay line between polarizer #2 and optical circulator CL2, we precisely increased the length of the fiber delay line to shift the center of pumping to *u*_c_ = −0.042, 0.45 and 0.93 in sequence while measuring the time differences between the counter-propagating optical pulses.

**Figure 6:**
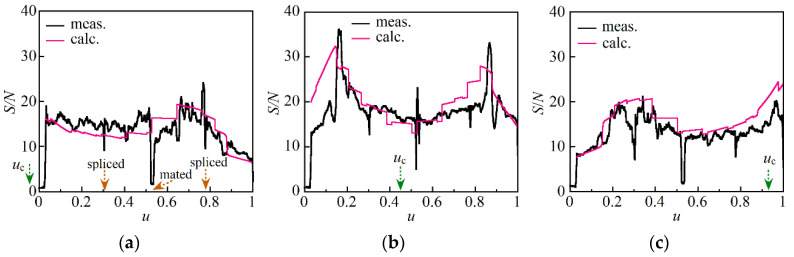
Distributions of S/N along a 1.35 m long optical fiber that were measured and calculated at (**a**) *u*_c_ = −0.042, (**b**) *u*_c_ = 0.45 and (**c**) *u*_c_ = 0.93, where the DFB LD was driven with current source A whose power spectral density is shown in Figure 5a. The scale of the horizontal axis is normalized in such a way that the output end of the fiber under test was unity. The rapid changes of S/N observed around *u* = 0.31, 0.53 and 0.78 were caused by fusion splicing and mating of the pieces of the optical fiber.

We drove the DFB LD with current source A. At each *u*_c_ value, we swept the tunable laser source at a rate of 0.5 nm/s 30 times and derived the magnitude of the Stokes light signal as a function of *z*. We calculated the mean value and standard deviation at every distance from the 30 reflectograms and plotted the distribution of the S/N in Figure 6 after changing the grids of the horizontal axis from *z* to *u*, which was defined as *u* = *z*/*z*_e_. Figure 6a–c show the S/N distributions obtained when we set *u*_c_ at −0.042, 0.45 and 0.93, respectively. Here it is noted that the Stokes light signals at the two spliced points and at one mated point of the APC connectors decreased so rapidly that the resultant S/N values were changed. The best S/N we achieved was only 36 at *u* = 0.16 and *u*_c_ = 0.45, where the fluctuations of the Stokes light signal were within 7% with respect to its mean level as shown in Figure 2a. The most values of S/N that we obtained by setting *u*_c_ at −0.042 and 0.93 ranged from 10 to 20.

Assuming that statistically independent random noise was superimposed on the Stokes light signal, we could increase the S/N by √*N* by calculating the mean value of *N* individual signals [24]. Therefore, we should acquire at least 30 beat signal waveforms by sweeping the tunable laser 30 times and calculate the mean reflectogram of the individual reflectograms to achieve an S/N of the order of 200 at every up-conversion frequency. We measured the S/N as a function of *u* when the DFB LD was driven with current source B.

**Figure 7:**
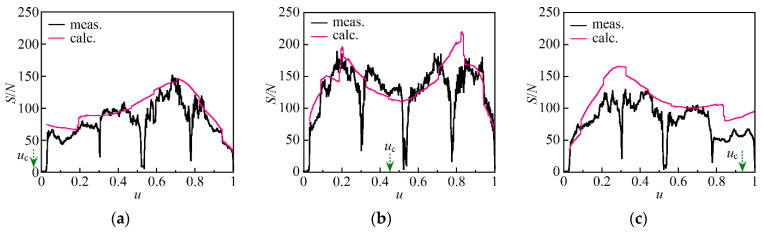
Distributions of S/N along the 1.35 m long optical fiber, which were measured and calculated at (**a**) *u*_c_ = −0.042, (**b**) *u*_c_ = 0.45 and (**c**) *u*_c_ = 0.93, where the DFB LD was driven with current source B whose power spectral density is shown in Figure 5b. The scale of the horizontal axis is normalized in such a way that the output end of the fiber under test was unity. The rapid changes of S/N observed around *u* = 0.31, 0.53 and 0.78 were caused by fusion splicing and mating of the pieces of the optical fiber.

The results are shown in Figure 7a–c when we set *u*_c_ at −0.042, 0.45 and 0.93, respectively. By comparing each pair in Figure 6 and Figure 7, e.g., Figure 6a and Figure 7a, it was clear that we could greatly increase the S/N merely by changing the current source from A to B. For example, the best S/N was increased to 190 at *u* = 0.18 and *u*_c_ = 0.45. This meant that a single sweep was sufficient to obtain a smooth reflectogram, which could be obtained only after sweeping the laser 30 times when we used current source A.

For comparison with the measured values, we calculated S/N as a function of *u* numerically using Equations (18) and (19). The theoretical S/N was given by the ratio of the Gaussian function to the square root of the variance *σ*^(2)^, where the Gaussian function could be regarded as unity when we tested the 1.35 m long optical fiber. This was concluded by calculating the value of *δν*_rms_ as follows. *δν*_rms_ was given by integrating *H*(*f*) over the interval (0, *f*_u_) with respect to *f* as described in Equation (10). Here we had already measured the power spectral density *H*(*f*) as shown in Figure 5a, where the sampling interval was Δ*f* = 0.0853 Hz and each sampling frequency was represented by *f_k_* = *k*Δ*f* (*k* = 0,1,2,…). The upper limit of integration was *f*_u_, which was the detection bandwidth and given by *f*_u_ = *τ*_e_*β*/2π. We obtained *τ*_e_ = 13.5 ns by substituting *z*_e_ = 139.8 cm and *n* = 1.45 into the relational expression of *τ*_e_ = 2*nz*_e_/*c*. Since we set the sweep speed of the tunable laser source at 0.5 nm/s at 1.55 μm, we obtained *β*/2π = 62.5 GHz/s. With these values we found that the upper limit of integration was *f*_u_ = 844 Hz.

We performed the numerical integration to obtain the value of *δν*_rms_ by approximating the integral of *H*(*f*) with respect to *f* as the sum of the areas *H_k_*Δ*f* of the equally-spaced rectangles under the curve *H*(*f*) for all values of *k* satisfying *f*_L_ < *f_k_* < *f*_u_, where the value of *H*(*f*) taken at *f_k_* is denoted as *H_k_*. In addition, we introduced the lower limit of integration at *f*_L_ = 1 Hz to avoid the effect of the environmental perturbations. Following the numerical calculation, we obtained *δν*_rms_ = 2.17 MHz and therefore we had Δ_rms_ = 1.36 × 10^7^ rad/s from the relational expression of Δ_rms_ = 2π*δν*_rms_. With the values of *τ*_e_ and Δ_rms_, we had Δ_rms_*τ*_e_ = 0.184 and thus we found that [Δ_rms_*τ*_e_(*u*_c_ − *u*)]^2^ < 0.0368 for all values of *u* and *u*_c_. The result meant that we could consider the Gaussian function as unity when we tested the 1.35 m long optical fiber.

We calculated *σ*^(2)^ as a function of *u* ranging from 0 to 1 in steps of 0.01, where *βτ*_e_^3^ = 9.66 × 10^−13^ s, *u*_i_ = 0.0294 and the value of *G*(*χ*) at *χ* was obtained from that of 2*πH*(*f*) at *f* = *χ*/2π. That is, at each value of *u* for *u*_i_ < *u* < (*u*_i_ + 1)/2 and *u*_i_ = 0.0294, we approximated the first integral in Equation (19) as the sum of 2π(*u*_c_ – *u* − *η_k_*)^2^*H_k_*Δ*η* for all values of *k* satisfying max{*f*_L_,(*u* − *u*_i_)*f*_u_} < *f_k_* < (1 − *u*)*f*_u_, where *η_k_* = *f_k_*/*f*_u_, Δ*η* = Δ*f*/*f*_u_ and max{,} returns the greater of two values. Similarly, the second integral in Equation (19) was approximated as the sum of 2π*η_k_*^2^*H_k_*Δ*η* for all values of *k* satisfying *f*_L_ < *f_k_* < (*u* − *u*_i_)*f*_u_. With the two numerical integrations we calculated the value of *σ*^(2)^ and the resultant S/N according to Equation (18), where the numerator was approximated as unity. At each value of *u* for (*u*_i_ + 1)/2 < *u* < 1, we performed the same numerical calculation of the integrals in Equation (A45) in Appendix D to obtain *σ*^(2)^ and thus S/N as a function of *u*.

By repeating these series of numerical integrations at *u*_c_ = −0.042, 0.45 and 0.93, we obtained S/N as a function of *u* as shown in Figure 6a–c, respectively. Similarly, we calculated S/N as a function of *u* when the DFB LD was driven with current source B. The results are shown in Figure 7a–c when we set *u*_c_ at *u*_c_ = −0.042, 0.45 and 0.93, respectively. The calculation showed that when we set *u*_c_ at 0.45 or near the center of the optical fiber under test, the distribution of S/N had peaks on both sides of the center, whereas one or both peaks were suppressed at *u*_c_ = −0.042 and 0.93, which was consistent with the measurement result obtained using either current source A or current source B. We should set *u*_c_ close to 0.5 to avoid the suppression of the peaks and achieve a higher S/N throughout the fiber. It is noted that the value of *u*_c_ automatically approaches 0.5 as the length of the optical fiber under test is increased to 10 m or more. This is because the distance to the center of the optical fiber is much longer than the distance to the original center of pumping which is located if we connect the input end directly to the output end of the fiber.

**Figure 8:**
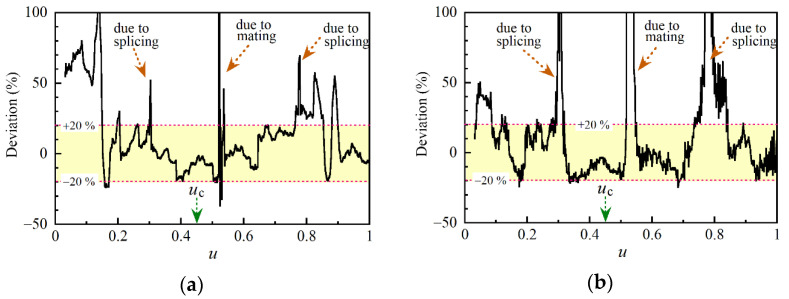
Difference between the calculated and measured S/N values in percent as a function of *u*. (**a**,**b**) were obtained from the data shown in Figure 6b and Figure 7b, respectively, where the center of pumping was located at *u*_c_ = 0.45. The range where the deviation was within 20% was highlighted in yellow in each figure. The rapid changes observed around *u* = 0.31, 0.53 and 0.78 were caused by fusion splicing and mating of the pieces of the single-mode fiber.

We plotted the difference between the calculated and measured S/N values in percent as a function of *u* in Figure 8, where (a) and (b) show the distributions calculated from the data shown in Figure 6b and Figure 7b, respectively. The optical fiber under test consisted of four pieces of the single-mode fiber which were fusion spliced at *u* = 0.31 and 0.78 and mated with APC connectors at 0.53, where the Stokes light signal decreased and thus the deviation changed rapidly. Excluding these parts, however, the differences were limited to within 20% in most parts of the fiber as highlighted in yellow in each figure. We derived the theoretical expression for S/N by imposing a condition whereby the absolute value of the complex amplitude of the acoustic wave was constant along the fiber, whereas its temporal phase was modulated by the frequency fluctuations of the pump light. In spite of the fact that we could not experimentally generate the Brillouin dynamic grating uniformly throughout the fiber, the calculated values were in good agreement with those measured in the 10 to 190 range as shown in Figure 6 and Figure 7. Therefore, we confirmed that the theoretical expression we derived could represent the actual S/N of the reflection measurement and that the main source which substantially determined the S/N in our experiment was technical noise from the current source used to drive the DFB LD. Although S/N fell rapidly to 70 at both ends of the fiber as shown in Figure 7b even when we used current source B, we will be able to increase it to the same level as the peak by using a different current source with much reduced technical noise.

**Figure 9:**
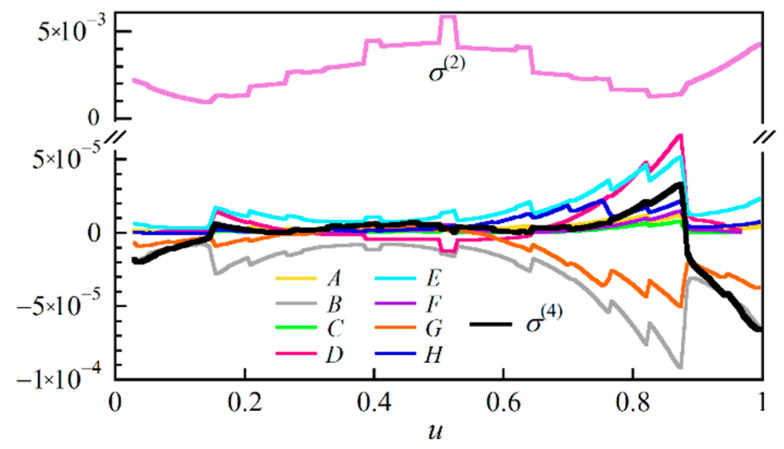
Numerical calculation of the constituent terms *A* to *H* of the correction term *σ*^(4)^ together with *σ*^(2)^ when current source A was used, where the center of pumping was located at *u*_c_ = 0.45. We calculated *σ*^(4)^ to improve the discrepancy between the measured and calculated data observed in Figure 6b.

Since the calculated values did not exactly agree with the measured ones obtained with either current source A or current source B, we calculated the correction term *σ*^(4)^ when using current source A at *u*_c_ = 0.45 by numerically calculating the constituent terms *A* through *H*, which are defined by Equations (A59) to (A66), while using the power spectral density shown in Figure 5a. The calculated values of *σ*^(2)^ and *σ*^(4)^ as a function of *u* are plotted in Figure 9 together with the change in each term. *σ*^(2)^ had a minimum value of 10^−3^ on both sides of the center of pumping, whereas *σ*^(4)^ had a maximum value of 3 × 10^−5^ at 0.1 < *u* < 0.9. Thus, we found that the correction term was at best 3% of *σ*^(2)^, and we could not bring the calculated values very much closer to the measured ones. The theoretical expressions for *σ*^(2)^ and *σ*^(4)^ were based on the expansion of the exponential function in the integrand of *I*(*t*) in a power series of Δ(*t*). The result showed that *σ*^(4)^ was negligibly smaller than *σ*^(2)^, and the first-order expansion was sufficient to calculate S/N as far as the reflection measurement of the 1.35 m long optical fiber was concerned.

Since the phases of the counter-propagating pump light waves always coincide with each other at the center of pumping, the temporal phase of the acoustic wave generated at the center is independent of the frequency fluctuations of the pump light waves. Considering that the phase of the Stokes light that was generated there was also unaffected by the fluctuations, it appears that the variance due to the frequency fluctuations become negligibly small and the resultant S/N should be at its maximum exactly at the center. Actually, however, when the center was located at *u*_c_ = 0.45, the S/N value was not at its maximum at the center and had noticeable peaks on both sides. The reflectometry method we employed decomposed the beat signal waveform into components with different frequencies and distributed the powers of the decomposed components along the fiber. When the frequency distribution of the beat signal waveform overlapped that of Δ(*t*), as in our experiment, the Stokes lights generated on either side of the center had components with the same frequency as that produced at the center via up and down frequency conversions by the phase modulation, which were superimposed on and recognized as the Stokes light generated at the center. This meant that even the signal detected at the center had larger fluctuations than we expected.

Planar lightwave circuits (PLCs) such as an 8 × 8 optical matrix switch and an arrayed-waveguide grating [7] have fiber pigtails several meters long, which are connected to their input and output ends with adhesive for practical applications. To investigate the possibility of using our reflectometer to diagnose them by means of strain distribution, we tested a 5 m long polarization-maintaining (PM) fiber. To excite the fast and slow axes of the PM fiber with the probe light and the counter-propagating pump lights, respectively, we spliced 250 µm buffered single-mode fibers with APC connectors to both ends of the PM fiber with a mechanical fusion splicer and mated the APC connectors to those of the fiber pigtails, which were attached to optical circulators CL1 and CL2. We inserted each 250 µm buffered single-mode fiber into an in-line polarization controller with a rotatable fiber squeezer mechanism to adjust the SOPs of the probe and pump lights propagating through the fiber.

**Figure 10:**
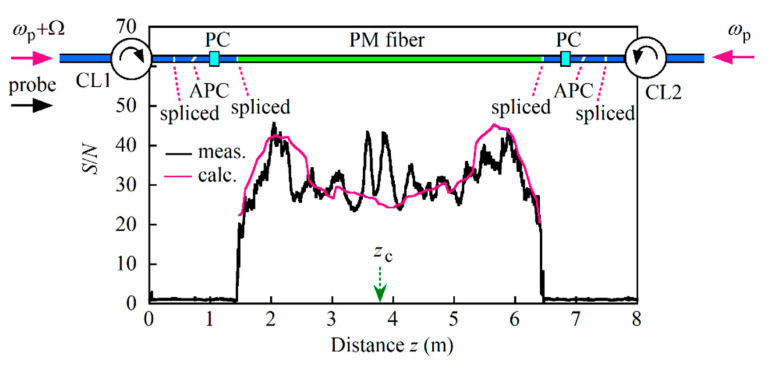
Comparison of the measured and calculated S/N along a 5 m long polarization-maintaining (PM) fiber, where the up-conversion frequency was 10.881 GHz and the DFB LD was driven with current source B whose power spectral density is shown in Figure 5b. Both ends of the PM fiber were spliced to 250 µm buffered single-mode fibers with APC connectors which were mated to those of the fiber pigtails of optical circulators CL1 and CL2. *z*_c_ (= 3.8 m) is the distance to the center of pumping. The fast and slow axes of the PM fiber were excited by the respective probe light and counter-propagating pump lights by using in-line polarization controllers (PCs). CL1 and CL2 are optical circulators. *ω*_p_ and *ω*_p_ + Ω are the angular frequencies of the counter-propagating pump light waves. APC: Angled physical contact.

After setting the up-conversion frequency at 10.881 GHz and changing to current source B, we measured 30 reflectograms from the 5 m long PM fiber and calculated the S/N distribution along the fiber as shown in Figure 10. As a result of the measurement range being extended from 1 to 5 m, the S/N was degraded to the 20 to 45 range. Considering that except around the center of pumping at *z*_c_ = 3.8 m the measured and calculated values agreed, we concluded that we needed to use a current source with greatly reduced noise to increase the S/N. Although not shown in this paper, we observed a background component in the beat signal waveform that undulated periodically during the frequency sweep and which we did not observe when we tested the 1.35 m long single-mode fiber. We changed to another PM fiber with a different cutoff wavelength, but we still observed such a component. Therefore, we believe that some fraction of the launched probe and pump lights propagated without being attenuated through the fiber in the cladding modes such as the modes in the stress-applying parts or between it and the core. There was a probability that the periodic change in the S/N that we observed around the center was produced by the light component, which propagated in the cladding modes and entered the balanced mixer to produce the fluctuated components by interference between it and the LO light.

**Figure 11:**
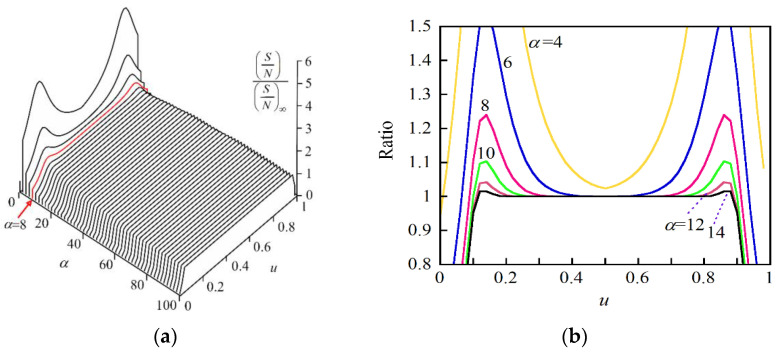
(**a**) Ratio of S/N to (S/N) _∞_ as functions of α and *u*. (**b**) Ratio of S/N to (S/N) _∞_ as a function of *u* when α was changed from 4 to 14 in steps of 2. α = *βτ*_e_/*δχ_e_*, where *β* is the sweep rate of the angular frequency of the tunable laser output, *τ*_e_ is the round-trip time from the origin at *z* = 0 to the output end of the fiber under test at *z* = *z*_e_ and thus *u* = *z*/*z*_e_. *δχ_e_* is the spectral half width at 1/*e* maximum of the Gaussian spectrum *G*(*χ*).

We investigated the specifications of the pump light wave required for achieving the S/N at 200 even when we tested a 5 m long PM fiber. When the power spectral density was a Gaussian function, we obtained the analytical solution to *σ*^(2)^, as detailed in Appendix F. We calculated the ratio of S/N to (S/N)_∞_ as functions of α and *u* using Equations (A81) and (A82) in Appendix F and plotted the result in Figure 11a. When *α* was close to 0, the ratio had the same two peaks as in Figure 6b and Figure 7b and then approached unity for almost all values of *u* as *α* increased. To observe the precise change in the ratio around *α* = 10, we plotted the ratio as a function of *u* by setting *α* at 4 to 14 in steps of 2 in Figure 11b. It was clear from the figure that at *α* = 8 the maximum deviation of the ratio from unity was only 23% in the 0.1 < *u* < 0.9 range and the deviation decreased rapidly as *α* increased from 8. Since a difference of around 20% was unavoidable between the calculated and measured data, as observed in Figure 8, we confirmed that we could regard the ratio as unity as long as *α* ≥ 8, where we could use the simple expression given by Equation (20).

**Figure 12:**
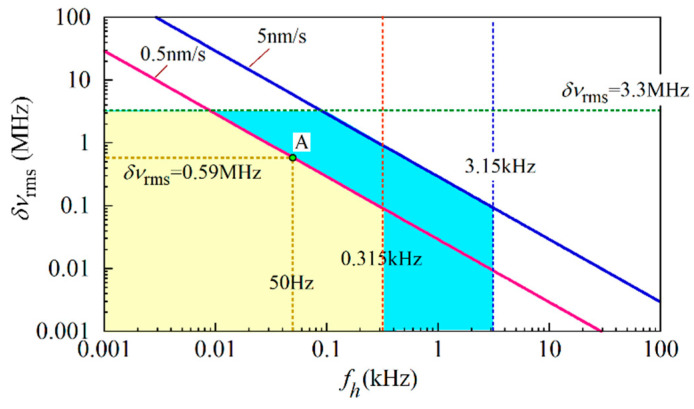
Domain highlighted in yellow where (*f*_h_, *δν*_rms_) satisfies the three conditions described by the relational expressions (21), (22) and (23) to achieve (*S*/*N*)_tgt_ = 200 at *τ*_e_ = 48.4 ns, *γ* = 62.5 GHz. The segment highlighted in blue is the domain extended by increasing the sweep rate to *γ* = 625 GHz/s. The red and blue solid lines show the boundary curves defined by *δν*_rms_ = 0.0293/*f*_h_ and *δν*_rms_ = 0.293/*f*_h_ in a log-log scale to satisfy the third expression (23) at 0.5 nm/s (= 62.5 GHz/s) and at 5 nm/s (= 625 GHz/s), respectively.

In the relational expressions of (21) and (22), *τ*_e_ was the round-trip time from the origin to the output end of the fiber under test and was given by *τ*_e_ =2*nL*/*c*, where we assumed that the fiber was long enough to allow *z*_i_ = 0. With the values *L* = 5 m and *n* = 1.45, we estimated *τ*_e_ to be 48.4 ns. By substituting *τ*_e_ = 48.4 ns and *γ* = 62.5 GHz (or 0.5 nm/s) into the right-hand sides of expressions (21) and (22), we obtained upper limits for *f*_h_ and *δν*_rms_ of 0.315 kHz and 3.3 MHz, respectively. In Figure 12 we plot red and green dotted lines, which are upper boundaries defined by *f*_h_ = 0.315 kHz and *δν*_rms_ = 3.3 MHz, respectively, in two-dimensional space with a (*f*_h_, *δν*_rms_) coordinate system. By substituting *γ* = 62.5 GHz and (*S*/*N*)_tgt_ = 200 into the right-hand side of the relational expression (23), we found that the point (*f*_h_, *δν*_rms_) should be included in the domain located below the red solid line, which was the boundary curve defined by *δν*_rms_ = 0.0293/*f*_h_ and was plotted in a log-log scale, where the units of *f*_h_ and *δν*_rms_ are kHz and MHz, respectively. Therefore, when we sweep the laser at the rate of *γ* = 62.5 GHz, the point (*f*_h_, *δν*_rms_) should be included in the allowable domain surrounded by the three lines highlighted in yellow in Figure 12 to achieve (*S*/*N*)_tgt_ = 200.

For example, when the spectral half width is reduced to *f*_h_ = 50 Hz by installing a low pass filter in the current source circuit, the rms frequency fluctuations should be equal to or lower than *δν*_rms_ = 0.59 MHz. This is determined by drawing a vertical line at *f*_h_ = 50 Hz and finding the coordinate of the point A, which is the intersection between the vertical line and the red solid line. When the spectral half width is much wider due to the potential difficulty involved in installing such a low pass filter, one way to achieve (*S*/*N*)_tgt_ is to sweep the tunable laser faster in such a way that the point (*f*_h_, *δν*_rms_) is included in the extended domain determined by the new sweep rate. For example, by letting *γ* = 625 GHz or 5 nm/s, the domain highlighted in blue is added as the allowable one so that the range of the spectral half width becomes ten times wider.

## 5. Discussion

This section describes the effect of the coherence time of the pump light on the S/N of the reflection measurement. When the counter-propagating pump light waves collided at *z* along the 1.35 m long optical fiber, the difference between the propagation times of the two pump light waves was given by |*τ*_c_ − *τ*| where *τ*_c_ = 2*nz*_c_/*c* and *τ* = 2*nz*/*c*, whose upper limit was *τ*_e_ = 13.5 ns for all possible combinations of (*τ*_c_, *τ*) satisfying 0 < *τ*_c_ < *τ*_e_ and 0 < *τ* < *τ*_e_. According to the data supplied by the manufacturer of the LD, the nominal spectral linewidth was of the order of 1 MHz, and so the coherence time of the pump light waves was estimated to be a few microseconds. That is, the coherence time was much longer than any possible values of the time differences between the pump light waves, and they were considered to be coherent with each other anywhere along the 1.35 m long optical fiber. In the frequency domain, the individual spectral components in the pump light waves should receive the same phase modulation from the technical noise and generate the same acoustic wave along the entire length of the fiber.

To observe the change in the Stokes light signal level as the fiber length increased, we spliced 250 µm buffered single-mode fibers with APC connectors to both ends of a 10 m long PM fiber and installed it as the DUT in the reflectometer. After testing the 10 m long PM fiber, we prepared a 40 m long PM fiber as the DUT and tested it again. Here we increased the carrier frequency for detecting the beat signal waveform to 300 kHz by driving the phase modulators PM1 and PM3 at *f*_0_ = 500 kHz and *f*_1_ = 800 kHz, respectively. This was because the maximum frequency of the beat signal waveform was increased to 25 kHz. In accordance with the higher detection frequency, we increased the carrier frequency of the unbalanced MZI, which we used to measure the power spectral density, to the same frequency at 300 kHz. As the fiber length increased, unfortunately the number of data needed for the numerical integration also increased, and this resulted in a longer calculation time and a fatal memory overflow. To avoid such results, we reduced the record length of the beat signal waveform to one-tenth and increased the sampling interval of the power spectral density to 0.853 Hz.

**Figure 13:**
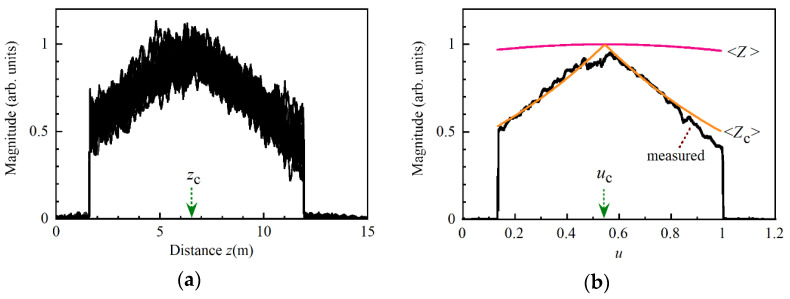
(**a**) Overwrite of 30 reflectograms from a 10 m long polarization-maintaining (PM) fiber. *z*_c_ (= 6.51 m) is the distance to the center of pumping. (**b**) Mean reflectogram (shown in black), which was derived from the 30 reflectograms shown in (**a**). <*Z*> is a plot of the Gaussian function defined by Equation (17) with Δ_rms_ = 3.74 × 10^6^ rad/s, *τ*_e_ = 115 ns and *u*_c_ = 0.545. <*Z*_c_> is a plot of Equation (28), which is the product of Gaussian and exponential functions where *δν*_L_ = 2 MHz.

We obtained 30 reflectograms from the 10 m long PM fiber and plotted them all and their mean reflectogram in Figure 13a,b, respectively. It was clear from the figures that all these reflectograms had peaks at the center of pumping and decayed as the position deviated from the center. In the first place, the mean Stokes light signal level <*Z*> should decrease due to the technical noise according to the Gaussian function as given by Equation (17).

We numerically integrated *H*(*f*) with respect to *f* using Equation (10) and obtained *δν*_rms_ = 0.60 MHz and thus Δ_rms_ = 3.74 × 10^6^ rad/s. With the value of Δ_rms_, *τ*_e_ = 115 ns and *u*_c_ = 0.545, we calculated <*Z*> as a function of *u* as drawn by the red solid curve in Figure 13b and found that the decay induced by the technical noise was too small to fit the measured signal change. We employed an external-cavity tunable laser source and a DFB LD source in our experiment and both light sources were capable of causing the signal decay. If the finite coherence time of the output light from the former laser was the dominant origin of the decay, each waveform in Figure 13a,b should have a peak at *z* = 0 and decay as the distance increases. This was because the optical path length difference between the LO light and the Stokes light generated at *z* = 0 was zero and increased with distance. Actually, we observed the peak at the center of pumping, which was consistent with the fact that the optical path length difference between the counter-propagating pump light waves was zero at the center and increased as the position deviated from it. Therefore, it was clear that the decay we observed was caused by the finite coherence time of the pump light waves.

Since we measured the frequency fluctuations due to the technical noise by using an unbalanced MZI with a short delay of *τ*_MZI_ *=* 8.912 ns, we considered that the individual spectral components of the pump light passed through the MZI coherently and produced the same signal change with time, and thus the resultant power spectral density did not contain the effect of the finite coherence time. In Section 2, we denoted the amplitude of the electric field of the LD output as *A*(*t*) = *A*_0_exp(− *iϕ*(*t*)), where *A*_0_ was a constant and *ϕ*(*t*) represented the phase change due to the technical noise. To introduce the effect of the finite coherence time, we express the amplitude as *A*(*t*) = *A*_0_*Ã*(*t*)exp(− *iϕ*(*t*)) and let *Ã*(*t*) be the amplitude fluctuation caused by the finite coherence time while keeping <|*Ã*(*t*)|^2^> = 1. Then the analytic signal represented by Equation (2) should be changed to *I*_c_(*t*) as follows:

(24) $$I_{c}(t)=\int_{-\infty }^{+\infty }r(\tau_{1})\tilde{A}\left(t-\frac{\tau_{1}}{2}\right){\tilde{A}}^{*}\left(t-\tau_{c}+\frac{\tau_{1}}{2}\right)e^{-i\Delta (t)(\tau_{c}-\tau_{1})}e^{-i\varpi \tau_{1}}\;d\tau_{1},$$

where *t* = (*ῶ* + *ω*_p_ − *ω*_1_)/*β*.

Considering that the fluctuation *Ã*(*t*) was statistically independent of the frequency fluctuations Δ(*t*) due to the technical noise, a simple way to incorporate the coherence effect into the reflection measurement is to approximate the fluctuating product *Ã*(*t* − *τ*_1_/2)*Ã* *(*t* − *τ*_c_ + *τ*_1_/2) in the integrand of Equation (24) by the ensemble average <*Ã*(*t* − *τ*_1_/2)*Ã* *(*t* − *τ*_c_ + *τ*_1_/2)>, which is denoted as *V*(*τ*_c_ − *τ*_1_) using the coherence function *V*(*τ*) defined by:

(25) $$V(\tau )=\int_{-\infty }^{+\infty }G_{p}\left(\omega_{p}+\varpi \right)e^{-i\varpi \tau }d\varpi .$$

*G*_p_(*ω*_p_ + *ῶ*) was the optical power spectrum of the pump light, and we assumed that the spectrum was normalized in such a way that ∫_−∞_^+∞^*G*_p_(*ω*_p_ + *ῶ*)*dῶ* = 1. Then the analytic signal *I*_c_(*t*) was approximated by:

(26) $$\langle I_{c}(t)\rangle =\int_{-\infty }^{+\infty }r(\tau_{1})V\left(\tau_{c}-\tau_{1}\right)e^{-i\Delta (t)(\tau_{c}-\tau_{1})}e^{-i\varpi \tau_{1}}\;d\tau_{1}.$$

Equation (26) means that the mean signal level <*Z*_c_>, the variance *σ*_c_^(2)^ and the correction term *σ*_c_^(4)^ including the coherence effect are obtained by replacing *r*(*τ*) with *r*(*τ*)*V*(*τ*_c_ − *τ*) in Equations (12), (16), and (A31)–(A38) in Appendix C, respectively.

Supposing that the pump light has a Lorentzian spectrum with a full width at half maximum of *δν*_L_, we obtain a real function of *V*(*τ*) = exp(− *πδν*_L_|*τ*|). By introducing a new function *U*(*u*) which is defined by:

(27) $$U(u)=V(\tau_{e}u)=e^{-\pi \delta \nu_{L}\tau_{e}\left|u\right|},$$

<*Z*_c_> and *σ*_c_^(2)^ for *u*_1_ < *u* < (*u*_1_ + 1)/2 are denoted as:

(28) $$\langle Z_{c}\rangle \approx e^{-{\left[\Delta_{\mathrm{rms}}\tau_{e}\left(u_{c}-u\right)\right]}^{2}}U^{2}\left(u_{c}-u\right),$$

(29) $$\begin{array}{l} \sigma_{c}{}^{\left(2\right)}=2\left(\tau_{e}^{3}\beta \right)U^{2}\left(u_{c}-u\right)\{\int_{u-u_{i}}^{1-u}U^{2}\left(u_{c}-u-\eta \right){\left(u_{c}-u-\eta \right)}^{2}G(\beta \tau_{e}\eta )\;d\eta \\ +\int_{0}^{u-u_{i}}{\left\{U\left(u_{c}-u-\eta \right)\left(u_{c}-u-\eta \right)-U\left(u_{c}-u+\eta \right)\left(u_{c}-u+\eta \right)\right\}}^{2}G(\beta \tau_{e}\eta )\;d\eta \}. \end{array}$$

To obtain the expression for (*u*_i_ + 1)/2 < *u* <1, in Equation (29) we should change the interval of the first integral to (1 − *u*, *u* − *u*_i_) and that of the second integral to (0, 1 − *u*), and change the sign of the variable *η* in the integrands. We estimated the *δν*_L_ of the employed LD to be 2.0 MHz by fitting the theoretical curve defined by Equation (28) with the measured mean reflectogram, as shown by an orange curve in Figure 13b.

**Figure 14:**
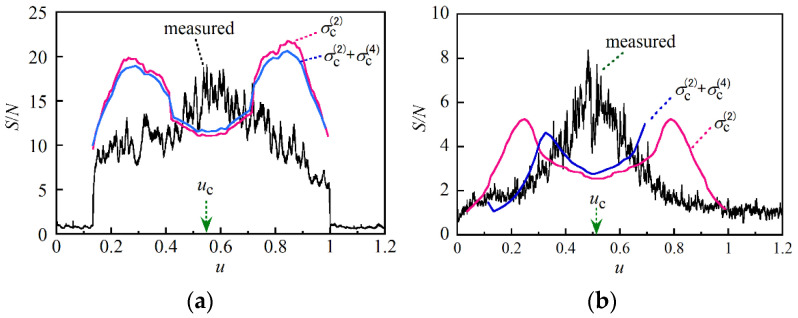
Comparison of measured and calculated S/N along (**a**) 10 m long and (**b**) 40 m long polarization-maintaining (PM) fibers. In each figure, the S/N distribution shown in black was obtained from distributions acquired by sweeping the tunable laser 30 times. The ratio <*Z*_c_>/√*σ*_c_^(2)^ as a function of *u* was calculated and plotted with a red line. The correction term *σ*_c_^(4)^ is expressed by the sum of the eight terms *A* to *H*, which were represented by Equations (A67) to (A74) in Appendix E, respectively. The ratio <*Z*_c_>/√(*σ*_c_^(2)^ + *σ*_c_^(2)^) as a function of *u* was calculated and plotted with a blue line. The employed parameters were (**a**) Δ_rms_ = 3.74 × 10^6^ rad/s, *τ*_e_ = 115 ns, *u*_c_ = 0.545. (**b**) Δ_rms_ = 5.65 × 10^6^ rad/s, *τ*_e_ = 402 ns, *u*_c_ = 0.515.

We calculated the S/N as a function of *u* from 30 reflectograms which are shown in Figure 13a and plotted the result in Figure 14a. The distribution had rapidly varying components, but the overall profile had a peak at the center of the pumping and decayed as the distance from the center increased. We calculated *σ*_c_^(2)^ and *σ*_c_^(4)^ as a function of *u* numerically by using Equation (29) and Equations (A67) to (A74) in Appendix E, respectively. Then we plotted the two distributions of S/N, which we obtained by substituting *σ*_c_^(2)^ and *σ*_c_^(2)^ + *σ*_c_^(4)^ into the denominator of Equation (18), as shown by the red and blue lines, respectively, in Figure 14a. Since the calculated values differed from the measured values by a factor of up to 2, it was clear that we could obtain the approximate S/N value by using the theoretical expression even when the length of the optical fiber approached the coherence length of the pump light and the resultant S/N was degraded. However, they still had peaks on both sides of the center of pumping and there was a noticeable discrepancy between the calculated and measured reflection profiles. Then we measured the reflectograms from the 40 m long PM fiber and plotted the S/N distribution together with those which we calculated using *σ*_c_^(2)^ and *σ*_c_^(2)^ + *σ*_c_^(4)^ in Figure 14b. The best S/N value was further degraded to 8, and the calculated distribution had still the same two peaks.

Since the correction term *σ*_c_^(4)^ did not lead to a fundamental solution for suppressing the side peaks, we concluded that the discrepancy was not caused by the approximation of the factor exp{− *i*Δ(*t*)(*τ*_c_−*τ*)} in the integrand of <*I*_c_(*t*)> as the power series expansion. In deriving the expressions for *σ*_c_^(2)^ and *σ*_c_^(2)^ + *σ*_c_^(4)^, we made one more approximation such that *ϕ*(*t* − *τ*/2) − *ϕ*(*t* − *τ*_c_ + *τ*/2) ≈ Δ(*t*)(*τ*_c_ − *τ*), where we theoretically showed that the approximation error was of the order of *π*^2^*f*_u_*δν*_rms_*τ*_e_^2^/2. When we tested the 40 m long PM fiber, we had *f*_u_ = 25 kHz, *δν*_rms_ = Δ_rms_/2π = 0.9 MHz and *τ*_e_ = 402 ns, and with these values we estimated the approximation error to be around 0.018 rad, and this meant that the approximation was still valid. Therefore, the origin of the noticeable discrepancy that we observed resulted not from the error due to the two kinds of approximations that we employed but from the simple method we introduced as an effect of the finite coherence time.

Returning to the basics of statistical averaging, therefore, we should calculate the mean value and variance of the absolute square of the Fourier inverse transform with two different kinds of statistically independent random processes such as Gaussian noise due to technical noise and AM and phase noise inherent in the laser diode [25,26]. If our reflectometry technique is to be applied to mid and long-range distributed strain sensing, the employed optical fiber will range in length from hundreds of meters to several kilometers. As described in our experiment, S/N will be degraded both by frequency fluctuations due to technical noise and by the finite coherence time unless we use an excellent DFB LD as the pump light source. Once we succeed in deriving the theoretical formula for S/N including the finite coherence time, we will be able to obtain the detailed specifications for the pump light source needed to achieve a high S/N even when we test an optical fiber several kilometers in length.

## 6. Conclusions

Signal-dependent speckle-like noise has been a serious factor in Brillouin-grating based coherent FMCW reflectometry. In this paper we theoretically and experimentally showed that the noise was generated by the frequency fluctuations of the pump light from the DFB LD. By assuming that the frequency of the pump light was modulated by the technical noise from the employed current source, we derived theoretical formulas for the mean value and the variance of the fluctuating power of the Stokes light, which contained the second and fourth-order moments of the frequency fluctuations. We adopted six experimental conditions for the reflectogram measurement where we used two current sources with different technical noises and set the center of pumping to the center and both sides of a 1.35 m long optical fiber under test. Under each condition we numerically calculated the mean value and variance along the optical fiber using the data for the power spectral density of the frequency fluctuations, and the resulting S/N distribution agreed with the measured distribution within 20% in most parts of the fiber. Although the reflectometry was proposed originally for the short-range diagnosis of miniaturized optical waveguides, our success shows that the reflectometry has a great potential for extending its application area to the middle and long-range fiber-optic distributed strain sensing.

## Figures and Tables

**Figure A1:**
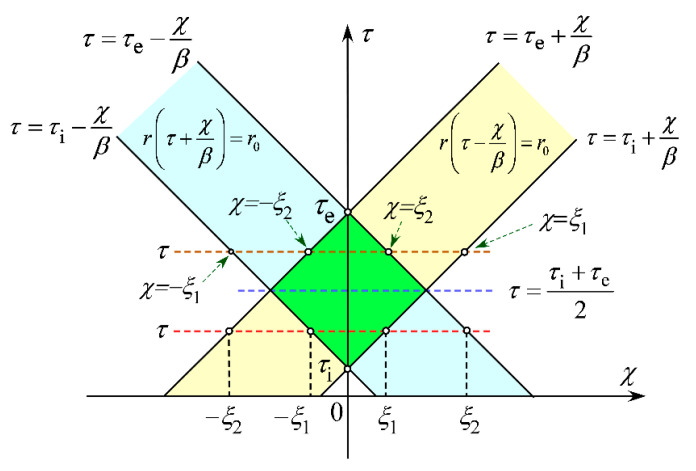
Domains of integration highlighted in yellow and light blue where the conditions of *r*(*τ* − *χ*/*β*) = *r*_0_ and *r*(*τ* + *χ*/*β*) = *r*_0_ are satisfied, respectively. *ξ*_1_ = *β*(*τ* − *τ*_i_) and *ξ*_2_ = *β*(*τ*_e_ − *τ*). Their overlapping domain is shaded in green. *β* is the sweep rate of the angular frequency of the employed tunable laser output, *τ*_i_ and *τ*_e_ are round trip times from the origin at *τ* = 0 to the input and output ends of the fiber under test, respectively.

**Figure A2:**
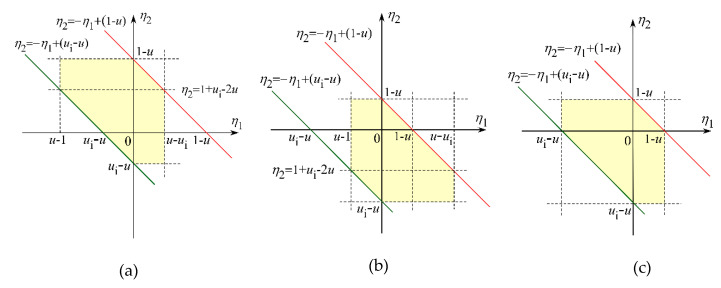
Domains highlighted in yellow for double integrals in (**a**) *G* for *u*_i_ < *u* < (*u*_i_ + 1)/2, (**b**) *G* for (*u*_i_ + 1)/2 < *u* < 1, and (**c**) *H* for *u*_i_ < *u* < 1. *u* = *τ*/*τ*_e_, *u*_c_ = *τ*_c_/*τ*_e_, *u*_i_ = *τ*_i_/*τ*_e_.

## Data Availability

Data sharing not applicable.

## Appendix A

The absolute square of the Fourier inverse transform of Equation (4) is expanded to:

(A1) $$\begin{array}{l} Z={\left|X^{\left(0\right)}+X^{\left(1\right)}+X^{\left(2\right)}+X^{\left(3\right)}+X^{\left(4\right)}\right|}^{2}\\ \;\;\;={\left|X^{\left(0\right)}\right|}^{2}\;\;\;\;\;\;\;\;\;\;\;\;\;\;\;\;\;\;\;\;\;\;\;\;\;\;\;\;\;\;\;\;\;\;\;\;\;\;\;\;\;\;\;\;\;\;\;(0th-order\;\mathrm{term})\\ \;\;\;+\left(X^{(0)*}X^{\left(1\right)}+c.c.\right)\;\;\;\;\;\;\;\;\;\;\;\;\;\;\;\;\;\;\;\;\;\;\;\;\;(1st-order\;\mathrm{term})\\ \;\;\;+{\left|X^{\left(1\right)}\right|}^{2}+\left(X^{(0)*}X^{\left(2\right)}+c.c.\right)\;\;\;\;\;\;\;\;(2nd-order\;\mathrm{term})\\ \;\;\;+X^{(0)*}X^{\left(3\right)}+X^{(1)*}X^{\left(2\right)}+c.c.\;\;\;\;\;\;\;(3rd-order\;\mathrm{term})\\ \;\;\;+{\left|X^{\left(2\right)}\right|}^{2}+\left(X^{(0)*}X^{\left(4\right)}+c.c.\right)\;\;\;\;\;\;\;\;(4th-order\;\mathrm{term})\\ \;\;\;+\mathrm{higher}-\mathrm{order}\;\mathrm{terms}, \end{array}$$

where the mean values of the first and third-order terms are zero because their integrands contain odd-order moments of Δ(*t*) which are equal to zero. For example:

(A2) $$\langle X^{(0)*}X^{\left(1\right)}\rangle =r^{*}(\tau )\frac{-i}{2\pi }\int_{-\infty }^{+\infty }\int_{-\infty }^{+\infty }r(\tau_{1})\langle \Delta (t_{1})\rangle (\tau_{c}-\tau_{1})e^{i\varpi_{1}\left(\tau -\tau_{1}\right)}\;d\tau_{1}d\varpi_{1}=0.$$

Since the mean values of the second and fourth-order terms are not zero, we add these values with the 0*th*-order term so that the mean value of Z, which is denoted as <Z>, is expressed by:

(A3) $$\langle Z\rangle ={\left|X^{\left(0\right)}\right|}^{2}+\langle {\left|X^{\left(1\right)}\right|}^{2}\rangle +\left(X^{(0)*}\langle X^{\left(2\right)}\rangle +c.c.\right)+\langle {\left|X^{\left(2\right)}\right|}^{2}\rangle +\left(X^{(0)*}\langle X^{\left(4\right)}\rangle +c.c.\right)\;$$

which is equivalent to Equation (7) by letting *X*^(0)^ = *r*. On the other hand, the residual component *δZ* in *Z* whose DC component is removed is represented by:

(A4) $$\delta Z=Y^{\left(1\right)}+Y_{1}^{\left(2\right)}+Y_{2}^{\left(2\right)}+Y^{\left(3\right)}+Y_{1}^{\left(4\right)}+Y_{2}^{\left(4\right)},$$

where:

(A5) $$Y^{\left(1\right)}=r*X^{\left(1\right)}+c.c.,$$

(A6) $$Y_{1}^{\left(2\right)}={\left|X^{\left(1\right)}\right|}^{2}-\langle {\left|X^{\left(1\right)}\right|}^{2}\rangle ,$$

(A7) $$Y_{2}^{\left(2\right)}=r*\left(X^{\left(2\right)}-\langle X^{\left(2\right)}\rangle \right)+c.c.,$$

(A8) $$Y^{\left(3\right)}=r*X^{\left(3\right)}+X^{\left(1\right)}*X^{\left(2\right)}+c.c.,$$

(A9) $$Y_{1}^{\left(4\right)}={\left|X^{\left(2\right)}\right|}^{2}-\langle {\left|X^{\left(2\right)}\right|}^{2}\rangle ,$$

(A10) $$Y_{2}^{\left(4\right)}=r^{*}\left(X^{\left(4\right)}-\langle X^{\left(4\right)}\rangle \right)+c.c.$$

It is noted that the variance of *Z* is given by <*δZ*^2^ >, which is Equation (8).

## Appendix B

From Equation (A5) we have <*Y*^(1)2^> = 2|*r*|^2^ <|*X*^(1)^|^2^> +(*r**^2^ <*X*^(1)2^> +c.c.), into each term of which we substitute the original expression for *X*^(1)^ given by Equation (5) at *k* = 1. First, we find that:

(A11) $$\langle {\left|X^{\left(1\right)}\right|}^{2}\rangle =\frac{1}{4\pi^{2}}\int r(\tau_{1})r^{*}(\tau_{2})\langle \Delta (t_{1})\Delta (t_{2})\rangle (\tau_{c}-\tau_{1})(\tau_{c}-\tau_{2})e^{i\varpi_{1}\left(\tau -\tau_{1}\right)}e^{-i\varpi_{2}\left(\tau -\tau_{2}\right)}\;d\tau_{1}d\varpi_{1}d\tau_{2}d\varpi_{2},$$

where the domain of the quadruple integral is (−∞, +∞)^4^ and *t*_1,2_ = (*ῶ*_1,2_ + *ω*_p_ − *ω*_1_)/*β*. The second-order moment of Δ(*t*) in the integrand of Equation (A11) is replaced with ∫_−∞_^+∞^ *G*(*χ*)exp{− *i*(*t*_1_−*t*_2_)*χ*}*dχ* according to Equation (9), and this expression is changed to the quintuple integral as:

(A12) $$\langle {\left|X^{\left(1\right)}\right|}^{2}\rangle =\frac{1}{4\pi^{2}}\int r(\tau_{1})r^{*}(\tau_{2})(\tau_{c}-\tau_{1})(\tau_{c}-\tau_{2})e^{i\varpi_{1}\left(\tau -\tau_{1}-\chi /\beta \right)}e^{i\varpi_{2}\left(\tau_{2}-\tau +\chi /\beta \right)}G(\chi )\;d\chi d\tau_{1}d\varpi_{1}d\tau_{2}d\varpi_{2}.$$

In Equation (A12) we take iterated integrals with respect to *ῶ*_1_ and *ῶ_2_* to give 2*πδ*(*τ* − *τ*_1_ − *χ*/*β*) and 2*πδ*(*τ*_2_ − *τ* + *χ*/*β*), respectively, where *δ*(*τ*) is the delta function. In the resultant triple integral including the delta functions, we repeat iterated integrals with respect to *τ*_1_ and *τ*_2_ to finally obtain:

(A13) $$\langle {\left|X^{\left(1\right)}\right|}^{2}\rangle ={\int_{-\infty }^{+\infty }\left|r\left(\tau -\frac{\chi }{\beta }\right)\right|}^{2}{\left(\tau_{c}-\tau +\frac{\chi }{\beta }\right)}^{2}G(\chi )\;d\chi .$$

Similarly, <*X*^(1)2^> is calculated as:

(A14) $$\begin{array}{l} \langle X^{\left(1\right)}{}^{2}\rangle =-\frac{1}{4\pi^{2}}\int r(\tau_{1})r(\tau_{2})\langle \Delta (t_{1})\Delta (t_{2})\rangle (\tau_{c}-\tau_{1})(\tau_{c}-\tau_{2})e^{i\varpi_{1}\left(\tau -\tau_{1}\right)}e^{i\varpi_{2}\left(\tau -\tau_{2}\right)}\;d\tau_{1}d\varpi_{1}d\tau_{2}d\varpi_{2}\\ \;\;\;\;\;\;\;\;\;\;\;\;\;\;=-\int_{-\infty }^{+\infty }r\left(\tau -\frac{\chi }{\beta }\right)r\left(\tau +\frac{\chi }{\beta }\right)\left[{\left(\tau_{c}-\tau \right)}^{2}-{\left(\frac{\chi }{\beta }\right)}^{2}\right]G(\chi )\;d\chi . \end{array}$$

From Equations (A13) and (A4), we obtain Equation (16).

## Appendix C

In this appendix we calculate the correction term *σ*^(4)^ defined by Equation (15). First, we substitute *Y*^(1)^ and *Y*^(3)^, which are defined by Equations (A5) and (A8), respectively, into the first term in Equation (15) to give:

(A15) $$\langle Y^{\left(1\right)}Y^{\left(3\right)}\rangle =r^{*2}\langle X^{\left(1\right)}X^{\left(3\right)}\rangle +{\left|r\right|}^{2}\langle X^{(1)*}X^{\left(3\right)}\rangle +r^{*}\langle {\left|X^{\left(1\right)}\right|}^{2}X^{\left(2\right)}\rangle +r\langle X^{(1)*2}X^{\left(2\right)}\rangle +c.c.$$

The first term in Equation (A15) contains the fourth-order moment of Δ(*t*) as:

(A16) $$\langle X^{\left(1\right)}X^{\left(3\right)}\rangle =\frac{1}{24\pi^{2}}\int r(\tau_{1})r(\tau_{3})\langle \Delta (t_{1})\Delta^{3}(t_{3})\rangle (\tau_{c}-\tau_{1}){\left(\tau_{c}-\tau_{3}\right)}^{3}e^{i\varpi_{1}\left(\tau -\tau_{1}\right)}e^{i\varpi_{3}\left(\tau -\tau_{3}\right)}\;d\tau_{1}d\varpi_{1}d\tau_{3}d\varpi_{3},$$

where *t*_1,3_ = (*ῶ*_1,3_ + *ω*_p_ − *ω*_1_)/*β*.

According to the moment theorem [21,22,23] we find that the moment in Equation (A16) is expanded to <Δ(*t*_1_)Δ^3^(*t*_3_)> = 3<Δ(*t*_1_)Δ(*t*_3_)><Δ^2^(*t*_1_)>, which is equal to 3Δ_rms_^2^∫_−∞_^+∞^ *G*(*χ*)exp {− *i*(*t*_1_ − *t*_3_)*χ*}*dχ* with Δ_rms_^2^ = <Δ^2^(*t*_1_)> and thus we obtain:

(A17) $$\langle X^{\left(1\right)}X^{\left(3\right)}\rangle =\frac{1}{8\pi^{2}}\Delta_{\mathrm{rms}}^{2}\int r(\tau_{1})r(\tau_{3})(\tau_{c}-\tau_{1}){\left(\tau_{c}-\tau_{3}\right)}^{3}e^{i\varpi_{1}\left(\tau -\tau_{1}-\chi /\beta \right)}e^{i\varpi_{3}\left(\tau -\tau_{3}+\chi /\beta \right)}G(\chi )\;d\chi d\tau_{1}d\varpi_{1}d\tau_{3}d\varpi_{3}.$$

We can reduce the quintuple integral to the following single one by taking the iterated integrals with respect to *ῶ*_1_ and *ῶ_3_* to produce delta functions, and then repeating the iterated integrals including the delta functions with respect to *τ*_1_ and *τ*_3_:

(A18) $$\langle X^{\left(1\right)}X^{\left(3\right)}\rangle =\frac{1}{2}\Delta_{\mathrm{rms}}^{2}\int r\left(\tau -\frac{\chi }{\beta }\right)r\left(\tau +\frac{\chi }{\beta }\right)\left(\tau_{c}-\tau +\frac{\chi }{\beta }\right){\left(\tau_{c}-\tau -\frac{\chi }{\beta }\right)}^{3}G(\chi )\;d\chi .$$

Similarly, we obtain the expression for the second term in Equation (A15) as:

(A19) $$\langle X^{(1)*}X^{\left(3\right)}\rangle =-\frac{1}{2}\Delta_{\mathrm{rms}}^{2}\int {\left|r\left(\tau +\frac{\chi }{\beta }\right)\right|}^{2}{\left(\tau_{c}-\tau -\frac{\chi }{\beta }\right)}^{4}G(\chi )\;d\chi .$$

The third term in Equation (A15) is written by a sextuple integral as:

(A20) $$\begin{array}{l} \langle {\left|X^{\left(1\right)}\right|}^{2}X^{\left(2\right)}\rangle =-\frac{1}{16\pi^{3}}\int r(\tau_{1})r^{*}(\tau_{2})r(\tau_{3})\langle \Delta (t_{1})\Delta (t_{2})\Delta^{2}(t_{3})\rangle (\tau_{c}-\tau_{1})(\tau_{c}-\tau_{2}){\left(\tau_{c}-\tau_{3}\right)}^{2}\\ \;\;\;\;\;\;\;\;\;\;\;\times e^{i\varpi_{1}\left(\tau -\tau_{1}\right)}e^{-i\varpi_{2}\left(\tau -\tau_{2}\right)}e^{i\varpi_{3}\left(\tau -\tau_{3}\right)}\;d\tau_{1}d\varpi_{1}d\tau_{2}d\varpi_{2}d\tau_{3}d\varpi_{3}. \end{array}$$

According to the moment theorem, the fourth-order moment in the integrand is expanded to:

(A21) $$\langle \Delta (t_{1})\Delta (t_{2})\Delta^{2}(t_{3})\rangle =\langle \Delta (t_{1})\Delta (t_{2})\rangle \langle \Delta^{2}(t_{3})\rangle +2\langle \Delta (t_{1})\Delta (t_{3})\rangle \langle \Delta (t_{2})\Delta (t_{3})\rangle ,$$

in which each term can be represented with *G*(*χ*).

By substituting Equation (A21) into Equation (A20) we find that:

(A22) $$\begin{array}{l} \langle {\left|X^{\left(1\right)}\right|}^{2}X^{\left(2\right)}\rangle =-\frac{1}{16\pi^{3}}\int r(\tau_{1})r^{*}(\tau_{2})r(\tau_{3})(\tau_{c}-\tau_{1})(\tau_{c}-\tau_{2}){\left(\tau_{c}-\tau_{3}\right)}^{2}e^{i\varpi_{1}\left(\tau -\tau_{1}\right)}e^{-i\varpi_{2}\left(\tau -\tau_{2}\right)}e^{i\varpi_{3}\left(\tau -\tau_{3}\right)}\\ \times \left[\Delta_{\mathrm{rms}}^{2}\int G(\chi )e^{-i\chi \left(\varpi_{1}-\varpi_{2}\right)/\beta }d\chi +2\int G(\chi_{1})G(\chi_{2})e^{-i\chi_{1}\left(\varpi_{1}-\varpi_{3}\right)/\beta }e^{-i\chi_{2}\left(\varpi_{2}-\varpi_{3}\right)/\beta }d\chi_{1}d\chi_{2}\right]\;d\tau_{1}d\varpi_{1}d\tau_{2}d\varpi_{2}d\tau_{3}d\varpi_{3}. \end{array}$$

By taking the iterated integrals with respect to *ῶ*_1_, *ῶ*_2_ and *ῶ_3_* and then by repeating the iterated integrals with respect to *τ*_1_, *τ*_2_ and *τ_3_*, in sequence, we finally obtain:

(A23) $$\begin{array}{l} \langle {\left|X^{\left(1\right)}\right|}^{2}X^{\left(2\right)}\rangle =-\frac{1}{2}r(\tau ){\left(\tau_{c}-\tau \right)}^{2}\Delta_{\mathrm{rms}}^{2}\int {\left|r\left(\tau -\frac{\chi }{\beta }\right)\right|}^{2}{\left(\tau_{c}-\tau +\frac{\chi }{\beta }\right)}^{2}G(\chi )\;d\chi \\ -\int r\left(\tau -\frac{\chi_{1}}{\beta }\right)r^{*}\left(\tau +\frac{\chi_{2}}{\beta }\right)r\left(\tau +\frac{\chi_{1}+\chi_{2}}{\beta }\right)\left(\tau_{c}-\tau +\frac{\chi_{1}}{\beta }\right)\left(\tau_{c}-\tau -\frac{\chi_{2}}{\beta }\right){\left(\tau_{c}-\tau -\frac{\chi_{1}+\chi_{2}}{\beta }\right)}^{2}\\ \;\;\;\;\;\;\;\times G(\chi_{1})G(\chi_{2})\;d\chi_{1}d\chi_{2}. \end{array}$$

Similarly, we obtain the expression for the last term in Equation (A15) as:

(A24) $$\begin{array}{l} \langle X^{(1)*2}X^{\left(2\right)}\rangle =\frac{1}{2}r(\tau ){\left(\tau_{c}-\tau \right)}^{2}\Delta_{\mathrm{rms}}^{2}\int r^{*}\left(\tau +\frac{\chi }{\beta }\right)r^{*}\left(\tau -\frac{\chi }{\beta }\right)\left[{\left(\tau_{c}-\tau \right)}^{2}-{\left(\frac{\chi }{\beta }\right)}^{2}\right]G(\chi )\;d\chi \\ +\int r^{*}\left(\tau +\frac{\chi_{1}}{\beta }\right)r^{*}\left(\tau +\frac{\chi_{2}}{\beta }\right)r\left(\tau +\frac{\chi_{1}+\chi_{2}}{\beta }\right)\left(\tau_{c}-\tau -\frac{\chi_{1}}{\beta }\right)\left(\tau_{c}-\tau -\frac{\chi_{2}}{\beta }\right){\left(\tau_{c}-\tau -\frac{\chi_{1}+\chi_{2}}{\beta }\right)}^{2}\\ \times G(\chi_{1})G(\chi_{2})\;d\chi_{1}d\chi_{2}. \end{array}$$

Substituting Equations (A18), (A19), (A23) and (A24) into Equation (A15) provides an explicit expression for <*Y*^(1)^*Y*^(3)^ >.

Next, we calculate the second term <(*Y*_1_^(2)^ + *Y*_2_^(2)^)^2^> in Equation (15). From the definitions of *Y*_1_^(2)^ and *Y*_2_^(2)^ given by Equations (A6), and (A7), respectively, we find that:

(A25) $$\begin{array}{l} \langle {\left(Y_{1}^{\left(2\right)}+Y_{2}^{\left(2\right)}\right)}^{2}\rangle =\left[\langle {\left|X^{\left(1\right)}\right|}^{4}\rangle -{\langle {\left|X^{\left(1\right)}\right|}^{2}\rangle }^{2}\right]+2{\left|r\right|}^{2}\left(\langle {\left|X^{\left(2\right)}\right|}^{2}\rangle -{\left|\langle X^{\left(2\right)}\rangle \right|}^{2}\right)\\ \;\;\;\;\;\;\;\;\;\;\;\;\;\;\;\;\;\;\;\;\;\;\;\;\;\;\;\;\;\;\;\;+\left[2r^{*}\left(\langle {\left|X^{\left(1\right)}\right|}^{2}X^{\left(2\right)}\rangle -\langle {\left|X^{\left(1\right)}\right|}^{2}\rangle \langle X^{\left(2\right)}\rangle \right)+c.c.\right]+\left[r^{*2}\left(\langle X^{(2)2}\rangle -{\langle X^{\left(2\right)}\rangle }^{2}\right)+c.c.\right]. \end{array}$$

In the same way as we derived the result for <*Y*^(1)^*Y*^(3)^ >, the individual terms in Equation (A25) are transformed into single or double integrals with respect to *χ* as follows:

(A26) $$\begin{array}{l} \langle {\left|X^{\left(1\right)}\right|}^{4}\rangle -{\langle {\left|X^{\left(1\right)}\right|}^{2}\rangle }^{2}\\ ={\left[\int {\left|r\left(\tau -\frac{\chi }{\beta }\right)\right|}^{2}{\left(\tau_{c}-\tau +\frac{\chi }{\beta }\right)}^{2}G(\chi )\;d\chi \right]}^{2}+{\left|\int r\left(\tau -\frac{\chi }{\beta }\right)r\left(\tau +\frac{\chi }{\beta }\right)\left[{\left(\tau_{c}-\tau \right)}^{2}-{\left(\frac{\chi }{\beta }\right)}^{2}\right]G(\chi )\;d\chi \right|}^{2}, \end{array}$$

(A27) $$\langle {\left|X^{\left(2\right)}\right|}^{2}\rangle -{\left|\langle X^{\left(2\right)}\rangle \right|}^{2}=\frac{1}{2}{\int \left|r\left(\tau -\frac{\chi_{1}+\chi_{2}}{\beta }\right)\right|}^{2}{\left(\tau_{c}-\tau +\frac{\chi_{1}+\chi_{2}}{\beta }\right)}^{4}G(\chi_{1})G(\chi_{2})\;d\chi_{1}d\chi_{2},$$

(A28) $$\begin{array}{l} \langle {\left|X^{\left(1\right)}\right|}^{2}X^{\left(2\right)}\rangle -\langle {\left|X^{\left(1\right)}\right|}^{2}\rangle \langle X^{\left(2\right)}\rangle =-\int r\left(\tau -\frac{\chi_{1}}{\beta }\right)r^{*}\left(\tau +\frac{\chi_{2}}{\beta }\right)r\left(\tau +\frac{\chi_{1}+\chi_{2}}{\beta }\right)\left(\tau_{c}-\tau +\frac{\chi_{1}}{\beta }\right)\\ \;\;\;\;\;\;\;\;\;\;\;\;\;\;\;\;\;\;\;\;\;\;\;\;\;\;\;\;\;\;\;\;\;\;\;\;\;\;\;\;\;\;\;\;\;\;\;\;\;\;\;\;\;\;\;\;\;\;\;\;\times \left(\tau_{c}-\tau -\frac{\chi_{2}}{\beta }\right){\left(\tau_{c}-\tau -\frac{\chi_{1}+\chi_{2}}{\beta }\right)}^{2}G(\chi_{1})G(\chi_{2})\;d\chi_{1}d\chi_{2}, \end{array}$$

(A29) $$\langle X^{(2)2}\rangle -{\langle X^{\left(2\right)}\rangle }^{2}=\frac{1}{2}\int r\left(\tau -\frac{\chi_{1}+\chi_{2}}{\beta }\right)r\left(\tau +\frac{\chi_{1}+\chi_{2}}{\beta }\right){\left[{\left(\tau_{c}-\tau \right)}^{2}-\left(\frac{\chi_{1}+\chi_{2}}{\beta }\right)\right]}^{2}G(\chi_{1})G(\chi_{2})\;d\chi_{1}d\chi_{2}.$$

Substituting Equations (A26) to (A29) into Equation (A25) leads to an explicit expression for the second term <(*Y*_1_^(2)^ + *Y*_2_^(2)^)^2^> in Equation (15).

To summarize the calculation results, the correction term *σ*^(4)^ is represented by the sum of the following eight terms *A* through *H*:

(A30) $$\sigma^{\left(4\right)}=A+B+C+D+E+F+G+H,$$

(A31) $$A={\left[\int {\left|r\left(\tau -\frac{\chi }{\beta }\right)\right|}^{2}{\left(\tau_{c}-\tau +\frac{\chi }{\beta }\right)}^{2}G(\chi )\;d\chi \right]}^{2},$$

(A32) $$B=-2{\left|r(\tau )\right|}^{2}\Delta_{\mathrm{rms}}^{2}\int {\left|r\left(\tau -\frac{\chi }{\beta }\right)\right|}^{2}{\left(\tau_{c}-\tau +\frac{\chi }{\beta }\right)}^{2}\left\{{\left(\tau_{c}-\tau \right)}^{2}+{\left(\tau_{c}-\tau +\frac{\chi }{\beta }\right)}^{2}\right\}G(\chi )\;d\chi ,$$

(A33) $$C={\left|\int r\left(\tau -\frac{\chi }{\beta }\right)r\left(\tau +\frac{\chi }{\beta }\right)\left[{\left(\tau_{c}-\tau \right)}^{2}-{\left(\frac{\chi }{\beta }\right)}^{2}\right]G(\chi )\;d\chi \right|}^{2},$$

(A34) $$D=r^{*}{}^{2}(\tau )\Delta_{\mathrm{rms}}^{2}\int r\left(\tau -\frac{\chi }{\beta }\right)r\left(\tau +\frac{\chi }{\beta }\right)\left[{\left(\tau_{c}-\tau \right)}^{2}-{\left(\frac{\chi }{\beta }\right)}^{2}\right]\left[{\left(\tau_{c}-\tau \right)}^{2}+{\left(\tau_{c}-\tau -\frac{\chi }{\beta }\right)}^{2}\right]G(\chi )\;d\chi +c.c.,$$

(A35) $$E={\left|r(\tau )\right|}^{2}{\int \left|r\left(\tau -\frac{\chi_{1}+\chi_{2}}{\beta }\right)\right|}^{2}{\left(\tau_{c}-\tau +\frac{\chi_{1}+\chi_{2}}{\beta }\right)}^{4}G(\chi_{1})G(\chi_{2})\;d\chi_{1}d\chi_{2},$$

(A36) $$F=\frac{1}{2}r^{*2}(\tau )\int r\left(\tau -\frac{\chi_{1}+\chi_{2}}{\beta }\right)r\left(\tau +\frac{\chi_{1}+\chi_{2}}{\beta }\right){\left[{\left(\tau_{c}-\tau \right)}^{2}-{\left(\frac{\chi_{1}+\chi_{2}}{\beta }\right)}^{2}\right]}^{2}G(\chi_{1})G(\chi_{2})\;d\chi_{1}d\chi_{2}+c.c.,$$

(A37) $$\begin{array}{l} G=-4r^{*}(\tau )\int r\left(\tau -\frac{\chi_{1}}{\beta }\right)r^{*}\left(\tau +\frac{\chi_{2}}{\beta }\right)r\left(\tau +\frac{\chi_{1}+\chi_{2}}{\beta }\right)\left(\tau_{c}-\tau +\frac{\chi_{1}}{\beta }\right)\left(\tau_{c}-\tau -\frac{\chi_{2}}{\beta }\right)\\ \;\;\;\;\;\;\;\;\;\;\;\;\;\;\;\;\;\;\;\;\;\;\;\;\;\;\;\;\times {\left(\tau_{c}-\tau -\frac{\chi_{1}+\chi_{2}}{\beta }\right)}^{2}G(\chi_{1})G(\chi_{2})\;d\chi_{1}d\chi_{2}+c.c., \end{array}$$

(A38) $$\begin{array}{l} H=2r(\tau )\int r^{*}\left(\tau +\frac{\chi_{1}}{\beta }\right)r^{*}\left(\tau +\frac{\chi_{2}}{\beta }\right)r\left(\tau +\frac{\chi_{1}+\chi_{2}}{\beta }\right)\left(\tau_{c}-\tau -\frac{\chi_{1}}{\beta }\right)\left(\tau_{c}-\tau -\frac{\chi_{2}}{\beta }\right)\\ \;\;\;\;\;\;\;\;\;\;\;\;\;\;\;\;\;\;\;\;\;\;\times {\left(\tau_{c}-\tau -\frac{\chi_{1}+\chi_{2}}{\beta }\right)}^{2}G(\chi_{1})G(\chi_{2})\;d\chi_{1}d\chi_{2}+c.c. \end{array}$$

## Appendix D

Here we transform the expression (16) for the second-order term *σ*^(2)^ into a simpler form under the condition that *r*(*τ*) = constant = *r*_0_ at *τ*_i_ ≤ *τ*≤*τ*_e_ and *r*(*τ*) = 0 elsewhere. In Figure A1, we show the domains of integration where *τ*_i_ ≤ *τ* − *χ*/*β* ≤ *τ*_e_ to satisfy *r*(*τ* − *χ*/*β*) = *r*_0_ and *τ*_i_ ≤ *τ* + *χ*/*β* ≤ *τ*_e_ to satisfy *r*(*τ* + *χ*/*β*) = *r*_0_, in yellow and light blue, respectively, in two-dimensional (*χ*, *τ*) space. The overlapped domain is shown in green. For *τ*_i_ < *τ*<(*τ*_i_ + *τ*_e_)/2, the interval of integration in the first term of Equation (16) should be included in the yellow or green domain, or (−*ξ*_2_, *ξ*_1_), which is decomposed into (−*ξ*_2_, −*ξ*_1_) and (−*ξ*_1_, *ξ*_1_), where *ξ*_1_ = *β*(*τ* − *τ*_i_) and *ξ*_2_ = *β*(*τ*_e_ − *τ*). Then we can write the first term as:

(A39) $$\mathrm{first}\;\mathrm{term}=2{\left|r_{0}\right|}^{4}\left(\int_{-\xi_{2}}^{-\xi_{1}}+\int_{-\xi_{1}}^{0}+\int_{0}^{\xi_{1}}\right){\left(\tau_{c}-\tau +\frac{\chi }{\beta }\right)}^{2}G(\chi )\;d\chi .$$

We change the sign of the variable *χ* in the first and second integrals in Equation (A39) to transform their intervals of integration to (*ξ*_1_, *ξ*_2_) and (0, *ξ*_1_), respectively, where *G*(*χ*) is an even function of *χ* so that *G*(−*χ*) = *G*(*χ*). Then the integrand of the second integral is (*τ*_c_ – *τ* − *χ*/*β*)^2^*G*(*χ*), which is added to that of the third integral to become 2{(*τ*_c_ − *τ*)^2^ + (*χ*/*β*)^2^}*G*(*χ*). We thus find that

(A40) $$\mathrm{first}\;\mathrm{term}=2{\left|r_{0}\right|}^{4}\left\{{\int_{\xi_{1}}^{\xi_{2}}\left(\tau_{c}-\tau -\frac{\chi }{\beta }\right)}^{2}G(\chi )\;d\chi +2\int_{0}^{\xi_{1}}\left[{\left(\tau_{c}-\tau \right)}^{2}+{\left(\frac{\chi }{\beta }\right)}^{2}\right]G(\chi )\;d\chi \right\}.$$

Since the integrand of the second term in Equation (16) contains the function *r*(*τ* − *χ*/*β*)*r*(*τ* + *χ*/*β*), the interval should be in the overlapping green domain in Figure A1, or −*ξ*_1_ < *χ* < *ξ*_1_, and thus we obtain:

(A41) $$\mathrm{second}\;\mathrm{term}=-2\times {\left|r_{0}\right|}^{4}\int_{-\xi_{1}}^{\xi_{1}}\left[{\left(\tau_{c}-\tau \right)}^{2}-{\left(\frac{\chi }{\beta }\right)}^{2}\right]G(\chi )\;d\chi =-4{\left|r_{0}\right|}^{4}\int_{0}^{\xi_{1}}\left[{\left(\tau_{c}-\tau \right)}^{2}-{\left(\frac{\chi }{\beta }\right)}^{2}\right]G(\chi )\;d\chi .$$

By adding Equations (A40) to (A41), we have:

(A42) $$\sigma^{\left(2\right)}=2{\left|r_{0}\right|}^{4}\left[{\int_{\xi_{1}}^{\xi_{2}}\left(\tau_{c}-\tau -\frac{\chi }{\beta }\right)}^{2}G(\chi )\;d\chi +4\int_{0}^{\xi_{1}}{\left(\frac{\chi }{\beta }\right)}^{2}G(\chi )\;d\chi \right].$$

For (*τ*_i_ + *τ*_e_)/2 < *τ*< *τ*_e_, it is clear from Figure A1 that the interval of integration of the first term in Equation (16) is (−*ξ*_2_, *ξ*_1_), which is decomposed to (−*ξ*_2_, *ξ*_2_) and (*ξ*_2_, *ξ*_1_), whereas that of the second term is (−*ξ*_2_, *ξ*_2_). This means the desired expressions for the first and second terms for (*τ*_i_ + *τ*_e_)/2 < *τ* < *τ*_e_ are obtained by changing between *ξ*_1_ with *ξ*_2_ and by changing the sign of *χ* in Equations (A40) and (A41). After performing this exchange process and adding the results, we obtain:

(A43) $$\sigma^{\left(2\right)}=2{\left|r_{0}\right|}^{4}\left[{\int_{\xi_{2}}^{\xi_{1}}\left(\tau_{c}-\tau +\frac{\chi }{\beta }\right)}^{2}G(\chi )\;d\chi +4\int_{0}^{\xi_{2}}{\left(\frac{\chi }{\beta }\right)}^{2}G(\chi )\;d\chi \right].$$

Finally, to facilitate the numerical calculation, we introduce a variable *u* and parameters *u*_c_ and *u*_i_, which are defined by *u* = *τ*/*τ*_e_, *u*_c_ = *τ*_c_/*τ*_e_ and *u*_i_ = *τ*_i_/*τ*_e_, change the variable of integration from *χ* to *η* defined by *η* = *χ*/(*βτ*_e_), and let |*r*_0_| = 1 in Equations (A42) and (A43). We thus find that:

(A44) $$\sigma^{\left(2\right)}=2\tau_{e}^{3}\beta \left[\int_{u-u_{i}}^{1-u}{\left(u_{c}-u-\eta \right)}^{2}G(\beta \tau_{e}\eta )\;d\eta +4\int_{0}^{u-u_{i}}\eta^{2}G(\beta \tau_{e}\eta )\;d\eta \right]\;\mathrm{for}\;u_{i}<u<\frac{u_{i}+1}{2}.$$

It is noted that by introducing the new variable *u*, the ranges defined by *τ*_i_ < *τ* < (*τ*_i_ + *τ*_e_)/2 and (*τ*_i_ + *τ*_e_)/2 < *τ* < *τ*_e_ are changed to those defined by *u*_i_ < *u*<(*u*_i_ + 1)/2, and (*u*_i_ + 1)/2 < *u* < 1, respectively. The expression of *σ*^(2)^ for (*u*_i_ + 1)/2 < *u* < 1 is obtained by changing the interval of the first integral to (1 − *u*, *u* − *u*_i_) and that of the second one to (0, 1 − *u*), and changing the sign of the variable *η* in the integrand of the first term. That is, we find that:

(A45) $$\sigma^{\left(2\right)}=2\tau_{e}^{3}\beta \left[\int_{1-u}^{u-u_{i}}{\left(u_{c}-u+\eta \right)}^{2}G(\beta \tau_{e}\eta )\;d\eta +4\int_{0}^{1-u}\eta^{2}G(\beta \tau_{e}\eta )\;d\eta \right]\;\mathrm{for}\;\frac{u_{i}+1}{2}<u<1.$$

## Appendix E

In Appendix C we have shown that the general formula for the correction term *σ*^(4)^ is obtained by adding the eight terms *A* to *H*. Here we derive the expressions for the individual terms for *τ*_e_ < *τ* < (*τ*_i_ + *τ*_e_)/2 by imposing a condition whereby *r*(*τ*) = constant = *r*_0_ at *τ*_i_ ≤ *τ* ≤*τ*_e_ and *r*(*τ*) = 0 elsewhere. It is clear that the expressions of *A* through *D* for (*τ*_i_ + *τ*_e_)/2 < *τ* < *τ*_e_ are obtained by changing between *ξ*_1_ and *ξ*_2_ and by changing the sign of the variable *χ* in the respective expressions for *τ*_i_ <*τ* < (*τ*_i_ + *τ*_e_)/2. Since the integrands of *A* and *B*, which are represented by Equations (A31) and (A32), respectively, have the same forms as the first term in Equation (16), we can straightforwardly express the results for *A* and *B* as:

(A46) $$A={\left|r_{0}\right|}^{4}{\left\{{\int_{\xi_{1}}^{\xi_{2}}\left(\tau_{c}-\tau -\frac{\chi }{\beta }\right)}^{2}G(\chi )\;d\chi +2\int_{0}^{\xi_{1}}\left[{\left(\tau_{c}-\tau \right)}^{2}+{\left(\frac{\chi }{\beta }\right)}^{2}\right]G(\chi )\;d\chi \right\}}^{2},$$

(A47) $$B=-2{\left|r_{0}\right|}^{4}\Delta_{\mathrm{rms}}^{2}\times \left\{\begin{array}{l} \;{\int_{\xi_{1}}^{\xi_{2}}\left(\tau_{c}-\tau -\frac{\chi }{\beta }\right)}^{2}\left[{\left(\tau_{c}-\tau \right)}^{2}+{\left(\tau_{c}-\tau -\frac{\chi }{\beta }\right)}^{2}\right]G(\chi )\;d\chi \\ +2{\left(\tau_{c}-\tau \right)}^{2}\int_{0}^{\xi_{1}}\left[{\left(\tau_{c}-\tau \right)}^{2}+{\left(\frac{\chi }{\beta }\right)}^{2}\right]G(\chi )\;d\chi \\ +\int_{0}^{\xi_{1}}\left[{\left(\tau_{c}-\tau -\frac{\chi }{\beta }\right)}^{4}+{\left(\tau_{c}-\tau +\frac{\chi }{\beta }\right)}^{4}\right]G(\chi )\;d\chi \end{array}\right\},$$

Considering that *C* and *D*, which are expressed by Equation (A33) and (A34), respectively, contain the same function *r*(*τ* + *χ*/*β*) *r* (*τ* − *χ*/*β*) in their integrands as the second term in Equation (16), we find that:

(A48) $$C=4{\left|r_{0}\right|}^{4}{\left\{\int_{0}^{\xi_{1}}\left[{\left(\tau_{c}-\tau \right)}^{2}-{\left(\frac{\chi }{\beta }\right)}^{2}\right]G(\chi )\;d\chi \right\}}^{2},\;$$

(A49) $$D=4{\left|r_{0}\right|}^{4}\Delta_{\mathrm{rms}}^{2}\int_{0}^{\xi_{1}}\left[{\left(\tau_{c}-\tau \right)}^{2}-{\left(\frac{\chi }{\beta }\right)}^{2}\right]\times \left[2{\left(\tau_{c}-\tau \right)}^{2}+{\left(\frac{\chi }{\beta }\right)}^{2}\right]G(\chi )\;d\chi .$$

By introducing new variables *χ*_3_ and *χ*_4_, which are defined by *χ*_3_ = *χ*_1_ + *χ*_2_ and *χ*_4_ = *χ*_2_, Equations (A35) and (A36) are transformed into:

(A50) $$E={\left|r(\tau )\right|}^{2}{\int \left|r\left(\tau -\frac{\chi_{3}}{\beta }\right)\right|}^{2}{\left(\tau_{c}-\tau +\frac{\chi_{3}}{\beta }\right)}^{4}W(\chi_{3})\;d\chi_{3},$$

(A51) $$F=\frac{1}{2}r^{*2}(\tau )\int r\left(\tau -\frac{\chi_{3}}{\beta }\right)r\left(\tau +\frac{\chi_{3}}{\beta }\right){\left[{\left(\tau_{c}-\tau \right)}^{2}-{\left(\frac{\chi_{3}}{\beta }\right)}^{2}\right]}^{2}W(\chi_{3})\;d\chi_{3}+c.c.,$$

where:

(A52) $$W(\chi_{3})=\int G(\chi_{3}-\chi_{4})G(\chi_{4})\;d\chi_{4}.$$

The domain for the double integral in Equations (A35) and (A36) are |*χ*_1_| < *βτ*_e_ and |*χ*_2_| < *βτ*_e_ in the (*χ*_1_, *χ*_2_) plane because the detection frequency at the balanced mixer is band-limited to within *βτ*_e_. Since the variable *χ*_4_ is equal to *χ*_2_, the interval of integration for *W*(*χ*_3_) is (−*βτ*_e_, *βτ*_e_). Considering that *W*(*χ*) is a real-valued and even function of *χ*, we find that Equations (A50) and (A51) are similar to Equations (A31) and (A33), respectively, and for *τ*_i_ <*τ* < (*τ*_i_ + *τ*_e_)/2 we obtain:

(A53) $$E=2{\left|r_{0}\right|}^{4}\left\{{\int_{0}^{\xi_{2}}\left(\tau_{c}-\tau -\frac{\chi }{\beta }\right)}^{4}W(\chi )\;d\chi +\int_{0}^{\xi_{1}}{\left(\tau_{c}-\tau +\frac{\chi }{\beta }\right)}^{4}W(\chi )\;d\chi \right\},$$

(A54) $$F=2{\left|r_{0}\right|}^{4}\int_{0}^{\xi_{1}}{\left[{\left(\tau_{c}-\tau \right)}^{2}-\left(\frac{\chi }{\beta }\right)\right]}^{2}W(\chi )\;d\chi ,$$

and find that the expressions for (*τ*_i_ + *τ*_e_)/2 < *τ* <*τ*_e_ are obtained by changing between *ξ*_1_ and *ξ*_2_ and by changing the sign of the variable *χ* in the respective expressions for *τ*_i_ < *τ* < (*τ*_i_ + *τ*_e_)/2.

From Equation (A37) for *G*, the double integral should be taken on the domain in the two-dimensional (*χ*_1_, *χ*_2_) plane, which satisfies the three conditions of −*ξ*_2_ ≤ *χ*_1_ ≤ *ξ*_1_, −*ξ*_1_ ≤ *χ*_2_ ≤ *ξ*_2_, and −*ξ*_1_ < *χ*_1_ + *χ*_2_ < *ξ*_2_, where we have:

(A55) $$G=-8{\left|r_{0}\right|}^{4}\int \left(\tau_{c}-\tau +\frac{\chi_{1}}{\beta }\right)\left(\tau_{c}-\tau -\frac{\chi_{2}}{\beta }\right){\left(\tau_{c}-\tau -\frac{\chi_{1}+\chi_{2}}{\beta }\right)}^{2}G(\chi_{1})G(\chi_{2})\;d\chi_{1}d\chi_{2}.$$

From Equation (A38) for *H*, the double integral:

(A56) $$H=4{\left|r_{0}\right|}^{4}\int \left(\tau_{c}-\tau -\frac{\chi_{1}}{\beta }\right)\left(\tau_{c}-\tau -\frac{\chi_{2}}{\beta }\right){\left(\tau_{c}-\tau -\frac{\chi_{1}+\chi_{2}}{\beta }\right)}^{2}G(\chi_{1})G(\chi_{2})\;d\chi_{1}d\chi_{2}$$

should be taken in the domain which is defined by –*ξ*_1_ ≤ *χ*_1_, *χ*_2_, *χ*_1_ + *χ*_2_ ≤*ξ*_2_.

In this appendix we derive the expressions for the terms *A* to *H*, which all contain the same factor |*r*_0_|^4^. The correction term *σ*^(4)^ is the sum of these terms so that the variance of *σ*^(2)^ + *σ*^(4)^ is also proportional to |*r*_0_|^4^, meaning that the standard deviation is proportional to |*r*_0_|^2^. Considering that the mean signal level is also proportional to|*r*_0_|^2^ as clearly seen from Equation (12), S/N, which is defined by the ratio of the mean signal level to the standard deviation, either no longer includes the factor *r*_0_, or it is independent of *r*_0_. From this independence, we redefine the individual terms *A* to *H* as the ones that are obtained by letting |*r*_0_| = 1 in their original expressions.

In the same way as in Appendix D, we introduce the variable *u* and parameters *u*_c_ and *u*_i_, which are defined by *u* = *τ*/*τ*_e_, *u*_c_ = *τ*_c_/*τ*_e_, and *u*_i_ = *τ*_i_/*τ*_e_, respectively. By changing the variable of integration from *χ* to *η* defined by *η* = *χ*/(*βτ*_e_), S/N for *u*_i_ < *u* < (*u*_i_ + 1)/2 is written as:

(A57) $$\frac{S}{N}=\frac{e^{-{\left[\Delta_{\mathrm{rms}}\tau_{e}\left(u_{c}-u\right)\right]}^{2}}}{\sqrt{\sigma^{\left(2\right)}+\sigma^{\left(4\right)}}},\;$$

where:

(A58) $$\sigma^{\left(4\right)}=A+B+C+D+E+F+G+H,$$

and where:

(A59) $$A={\left(\tau_{e}^{3}\beta \right)}^{2}{\left\{\int_{u-u_{i}}^{1-u}{\left(u_{c}-u-\eta \right)}^{2}G(\beta \tau_{e}\eta )\;d\eta +2\int_{0}^{u-u_{1}}\left[{\left(u_{c}-u\right)}^{2}+\eta^{2}\right]G(\beta \tau_{e}\eta )\;d\eta \right\}}^{2},$$

(A60) $$B=-2\Delta_{\mathrm{rms}}^{2}\left(\tau_{e}^{5}\beta \right)\times \left\{\begin{array}{l} \;\int_{u-u_{i}}^{1-u}{\left(u_{c}-u-\eta \right)}^{2}\left[{\left(u_{c}-u\right)}^{2}+{\left(u_{c}-u-\eta \right)}^{2}\right]G(\beta \tau_{e}\eta )\;d\eta \\ +2\;{\left(u_{c}-u\right)}^{2}\int_{0}^{u-u_{i}}\left[{\left(u_{c}-u\right)}^{2}+\eta^{2}\right]G(\beta \tau_{e}\eta )\;d\eta \\ +\int_{0}^{u-u_{i}}\left[{\left(u_{c}-u-\eta \right)}^{4}+{\left(u_{c}-u+\eta \right)}^{4}\right]G(\beta \tau_{e}\eta )\;d\eta \end{array}\right\},$$

(A61) $$C=4{\left(\tau_{e}^{3}\beta \right)}^{2}{\left\{\int_{0}^{u-u_{i}}\left[{\left(u_{c}-u\right)}^{2}-\eta^{2}\right]G(\beta \tau_{e}\eta )\;d\eta \right\}}^{2},$$

(A62) $$D=4\Delta_{\mathrm{rms}}^{2}\left(\tau_{e}^{5}\beta \right)\int_{0}^{u-u_{i}}\left[{\left(u_{c}-u\right)}^{2}-\eta^{2}\right]\times \left[2\;{\left(u_{c}-u\right)}^{2}+\eta^{2}\right]G(\beta \tau_{e}\eta )\;d\eta ,$$

(A63) $$E=2\left(\tau_{e}^{5}\beta \right)\left\{\int_{0}^{1-u}{\left(u_{c}-u-\eta \right)}^{4}W(\beta \tau_{e}\eta )\;d\eta +\int_{0}^{u-u_{i}}{\left(u_{c}-u+\eta \right)}^{4}W(\beta \tau_{e}\eta )\;d\eta \right\},$$

(A64) $$F=2\left(\tau_{e}^{5}\beta \right)\int_{0}^{u-u_{i}}{\left[{\left(u_{c}-u\right)}^{2}-\eta^{2}\right]}^{2}W(\beta \tau_{e}\eta )\;d\eta ,$$

(A65) $$G=-8{\left(\tau_{e}^{3}\beta \right)}^{2}\int \left(u_{c}-u+\eta_{1}\right)\left(u_{c}-u-\eta_{2}\right){\left(u_{c}-u-\eta_{1}-\eta_{2}\right)}^{2}G(\beta \tau_{e}\eta_{1})G(\beta \tau_{e}\eta_{2})\;d\eta_{1}d\eta_{2},$$

(A66) $$H=4{\left(\tau_{e}^{3}\beta \right)}^{2}\int \left(u_{c}-u-\eta_{1}\right)\left(u_{c}-u-\eta_{2}\right){\left(u_{c}-u-\eta_{1}-\eta_{2}\right)}^{2}G(\beta \tau_{e}\eta_{1})G(\beta \tau_{e}\eta_{2})\;d\eta_{1}d\eta_{2}.$$

The expressions of *A* through *F* for (*u*_i_ + 1)/2 < *u* < 1 are obtained by changing between 1 − *u* and *u* − *u*_i_ and by changing the sign of the variable *η* in the respective expressions for *u*_i_ < *u* < (*u*_i_ + 1)/2. The domains for the double integral in Equation (A65) for *G* are highlighted in yellow in (a) and (b) in the two-dimensional (*η*_1_, *η*_2_) plane in Figure A2 for *u*_i_ < *u* <(*u*_i_ + 1)/2, and for (*u*_i_ + 1)/2 < *u* < 1, respectively. On the other hand, the domain for the double integral in Equation (A66) for *H* is highlighted in yellow in (c) in Figure A2, which can be used for all values of *u* for *u*_i_ < *u* < 1.

The correction term *σ*_c_^(4)^ including the coherence effect are obtained by replacing *r*(*τ*) with *r*(*τ*)*V*(*τ*_c_ − *τ*) in the constituent terms represented by Equations (A31) to (A38). Assuming that the coherence function *V*(*τ*) defined by Equation (25) is a real-valued function, the constituent terms *A* to *H* represented by Equations (A59) to (A66) should be changed to the following expressions for *u*_i_ < *u* < (*u*_i_ + 1)/2 using the function *U*(*u*) which is defined by *U*(*u*) = *V*(*τ*_e_*u*):

(A67) $$A={\left(\tau_{e}^{3}\beta \right)}^{2}{\left\{\begin{array}{l} \int_{0}^{1-u}U^{2}\left(u_{c}-u-\eta \right){\left(u_{c}-u-\eta \right)}^{2}G(\beta \tau_{e}\eta )\;d\eta \\ +\int_{0}^{u-u_{i}}U^{2}\left(u_{c}-u+\eta \right){\left(u_{c}-u+\eta \right)}^{2}G(\beta \tau_{e}\eta )\;d\eta \end{array}\right\}}^{2},$$

(A68) $$B=-2\Delta_{\mathrm{rms}}^{2}\left(\tau_{e}^{5}\beta \right)U^{2}\left(u_{c}-u\right)\times \left\{\begin{array}{l} \;\int_{0}^{1-u}U^{2}\left(u_{c}-u-\eta \right){\left(u_{c}-u-\eta \right)}^{2}\left[{\left(u_{c}-u\right)}^{2}+{\left(u_{c}-u-\eta \right)}^{2}\right]G(\beta \tau_{e}\eta )\;d\eta \\ +\int_{0}^{u-u_{i}}U^{2}\left(u_{c}-u+\eta \right){\left(u_{c}-u+\eta \right)}^{2}\left[{\left(u_{c}-u\right)}^{2}+{\left(u_{c}-u+\eta \right)}^{2}\right]G(\beta \tau_{e}\eta )\;d\eta \end{array}\right\},$$

(A69) $$C=4{\left(\tau_{e}^{3}\beta \right)}^{2}{\left\{\int_{0}^{u-u_{i}}U\left(u_{c}-u-\eta \right)U\left(u_{c}-u+\eta \right)\left[{\left(u_{c}-u\right)}^{2}-\eta^{2}\right]G(\beta \tau_{e}\eta )\;d\eta \right\}}^{2},$$

(A70) $$\begin{array}{l} D=4\Delta_{\mathrm{rms}}^{2}\left(\tau_{e}^{5}\beta \right)U^{2}\left(u_{c}-u\right)\int_{0}^{u-u_{i}}U\left(u_{c}-u-\eta \right)U\left(u_{c}-u+\eta \right)\left[{\left(u_{c}-u\right)}^{2}-\eta^{2}\right]\\ \;\;\;\;\;\;\;\;\;\;\;\;\;\;\;\;\;\;\;\;\;\;\;\;\;\;\;\;\;\;\;\;\;\;\;\;\;\;\;\;\;\;\;\;\;\;\;\;\;\;\;\;\;\;\;\;\;\;\;\times \left[2{\left(u_{c}-u\right)}^{2}+\eta^{2}\right]G(\beta \tau_{e}\eta )\;d\eta , \end{array}$$

(A71) $$E=2\left(\tau_{e}^{5}\beta \right)U^{2}\left(u_{c}-u\right)\left\{\begin{array}{l} \int_{0}^{1-u}U^{2}\left(u_{c}-u-\eta \right){\left(u_{c}-u-\eta \right)}^{4}W(\beta \tau_{e}\eta )\;d\eta \\ +\int_{0}^{u-u_{i}}U^{2}\left(u_{c}-u+\eta \right){\left(u_{c}-u+\eta \right)}^{4}W(\beta \tau_{e}\eta )\;d\eta \end{array}\right\},$$

(A72) $$F=2\left(\tau_{e}^{5}\beta \right)U^{2}\left(u_{c}-u\right)\int_{0}^{u-u_{i}}U\left(u_{c}-u-\eta \right)U\left(u_{c}-u+\eta \right){\left[{\left(u_{c}-u\right)}^{2}-\eta^{2}\right]}^{2}W(\beta \tau_{e}\eta )\;d\eta ,$$

(A73) $$\begin{array}{l} G=-8{\left(\tau_{e}^{3}\beta \right)}^{2}U\left(u_{c}-u\right)\int U\left(u_{c}-u+\eta_{1}\right)U\left(u_{c}-u-\eta_{2}\right)U\left(u_{c}-u-\eta_{1}-\eta_{2}\right)\\ \;\;\;\;\;\;\;\;\;\;\;\;\;\;\;\;\;\;\;\times \left(u_{c}-u+\eta_{1}\right)\left(u_{c}-u-\eta_{2}\right){\left(u_{c}-u-\eta_{1}-\eta_{2}\right)}^{2}G(\beta \tau_{e}\eta_{1})G(\beta \tau_{e}\eta_{2})\;d\eta_{1}d\eta_{2}, \end{array}$$

(A74) $$\begin{array}{l} H=4{\left(\tau_{e}^{3}\beta \right)}^{2}U\left(u_{c}-u\right)\int U\left(u_{c}-u-\eta_{1}\right)U\left(u_{c}-u-\eta_{2}\right)U\left(u_{c}-u-\eta_{1}-\eta_{2}\right)\\ \;\;\;\;\;\;\;\;\;\;\;\;\;\;\;\;\times \left(u_{c}-u-\eta_{1}\right)\left(u_{c}-u-\eta_{2}\right){\left(u_{c}-u-\eta_{1}-\eta_{2}\right)}^{2}G(\beta \tau_{e}\eta_{1})G(\beta \tau_{e}\eta_{2})\;d\eta_{1}d\eta_{2}. \end{array}$$

## Appendix F

Here we calculate S/N when the power spectral density is Gaussian. We assume that the fiber under test is long enough for us to approximate *u*_i_ as zero. We substitute *G*(*χ*) = *G*_0_exp[− (*χ*/*δχ_e_*)^2^] into Equation (19), change the integral variable from *η* to *t* = *αη* with *α* = *βτ*_e_/*δχ_e_* and let *u*_i_ = 0 to find that:

(A75) $$\;\sigma^{\left(2\right)}=2G_{0}\tau_{e}^{2}\delta \chi_{e}\left\{\int_{\alpha u}^{\alpha (1-u)}{\left(u_{c}-u-t/\alpha \right)}^{2}e^{-t^{2}}dt+4\int_{0}^{\alpha u}{\left(t/\alpha \right)}^{2}e^{-t^{2}}dt\right\}\;\mathrm{for}\;0<u<\frac{1}{2},$$

where we used *t* as the integral variable in this appendix only. By expanding the polynomial function of *t* in the integrand of the first term, we obtain:

(A76) $$\;\sigma^{\left(2\right)}=2G_{0}\tau_{e}^{2}\delta \chi_{e}\left\{{\left(u_{c}-u\right)}^{2}\int_{\alpha u}^{\alpha (1-u)}e^{-t^{2}}dt-\frac{2(u_{c}-u)}{\alpha }\int_{\alpha u}^{\alpha (1-u)}te^{-t^{2}}dt+\frac{1}{\alpha^{2}}\int_{\alpha u}^{\alpha (1-u)}t^{2}e^{-t^{2}}dt+\frac{4}{\alpha^{2}}\int_{0}^{\alpha u}t^{2}e^{-t^{2}}dt\right\}.$$

We can easily obtain the following list for the finite integrals of the Gaussian function [27]:

(A77) $$\int_{0}^{x}e^{-t^{2}}dt=\frac{\sqrt{\pi }}{2}\mathrm{erf}(x),\;\int_{0}^{x}te^{-t^{2}}dt=\frac{1}{2}\left(1-e^{-x^{2}}\right),\;\int_{0}^{x}t^{2}e^{-t^{2}}dt=-\frac{x}{2}e^{-x^{2}}+\frac{\sqrt{\pi }}{4}\mathrm{erf}(x)\;,$$

where erf(*x*) is the error function of *x*. From the list we can calculate explicitly the integrals to find that:

(A78) $$\;\sigma^{\left(2\right)}=2G_{0}\tau_{e}^{2}\delta \chi_{e}\left\{\begin{array}{l} \;\left[{\left(u_{c}-u\right)}^{2}+\frac{1}{2\alpha^{2}}\right]\frac{\sqrt{\pi }}{2}\mathrm{erf}\left(\alpha (1-u)\right)+\left[-{\left(u_{c}-u\right)}^{2}+\frac{3}{2\alpha^{2}}\right]\frac{\sqrt{\pi }}{2}\mathrm{erf}\left(\alpha u\right)\\ +\frac{1}{\alpha }\left(u_{c}-\frac{u}{2}-\frac{1}{2}\right)e^{-\alpha^{2}{\left(1-u\right)}^{2}}-\frac{1}{\alpha }\left(\frac{u}{2}+u_{c}\right)e^{-\alpha^{2}u^{2}}\;\;\;\;\;\;\;\;\;\;\mathrm{for}\;0<u<\frac{1}{2}. \end{array}\right\}$$

when α > >1, both error functions in Equation (A49) approach unity as long as *u* ≠ 0 and *u*≠1, and thus *σ*^(2)^ at α > >1 is:

(A79) $$\;\sigma_{\infty }^{\left(2\right)}=\frac{2G_{0}\tau_{e}^{2}\delta \chi_{e}\sqrt{\pi }}{\alpha^{2}}.$$

Similarly, we obtain the expression *σ*^(2)^ as:

(A80) $$\;\sigma^{\left(2\right)}=2G_{0}\tau_{e}^{2}\delta \chi_{e}\left\{\begin{array}{l} \;\left[-{\left(u_{c}-u\right)}^{2}+\frac{3}{2\alpha^{2}}\right]\frac{\sqrt{\pi }}{2}\mathrm{erf}\left(\alpha (1-u)\right)+\left[{\left(u_{c}-u\right)}^{2}+\frac{1}{2\alpha^{2}}\right]\frac{\sqrt{\pi }}{2}\mathrm{erf}\left(\alpha u\right)\\ +\frac{1}{\alpha }\left(u_{c}+\frac{u}{2}-\frac{3}{2}\right)e^{-\alpha^{2}{\left(1-u\right)}^{2}}+\frac{1}{\alpha }\left(\frac{u}{2}-u_{c}\right)e^{-\alpha^{2}u^{2}}\;\;\;\;\;\;\;\;\;\;\;\mathrm{for}\;\frac{1}{2}<u<1, \end{array}\right\}$$

from which *σ*^(2)^ at α > >1 is proved to have the same expression as that for 0 < *u* < 1/2. From Equation (16), S/N is expressed by *S*/*N* = exp{− [Δ_rms_*τ*_e_(*u*_c_−*u*)]^2^}/√*σ*^(2)^, whereas S/N at α > >1 is (*S*/*N*)_∞_ = exp{− [Δ_rms_*τ*_e_(*u*_c_−*u*)]^2^}/√*σ_∞_*^(2)^. Then their ratio is given as:

(A81) $$\frac{S}{N}/{\left(\frac{S}{N}\right)}_{\infty }=\frac{1}{\sqrt{\Gamma_{\mathrm{SNR}}}},$$

where:

(A82) $$\Gamma_{\mathrm{SNR}}=\frac{\alpha^{2}}{\sqrt{\pi }}\left\{ \begin{array}{l} \;\left[{\left(u_{c}-u\right)}^{2}+\frac{1}{2\alpha^{2}}\right]\frac{\sqrt{\pi }}{2}\mathrm{erf}\left(\alpha (1-u)\right)+\left[-{\left(u_{c}-u\right)}^{2}+\frac{3}{2\alpha^{2}}\right]\frac{\sqrt{\pi }}{2}\mathrm{erf}\left(\alpha u\right)\\ +\frac{1}{\alpha }\left(u_{c}-\frac{u}{2}-\frac{1}{2}\right)e^{-\alpha^{2}{\left(1-u\right)}^{2}}-\frac{1}{\alpha }\left(\frac{u}{2}+u_{c}\right)e^{-\alpha^{2}u^{2}}\;\;\;\;\;\;\;\;\;\;\;\;\;\;\;\;\mathrm{for}\;0<u<\frac{1}{2},\\ \;\left[-{\left(u_{c}-u\right)}^{2}+\frac{3}{2\alpha^{2}}\right]\frac{\sqrt{\pi }}{2}\mathrm{erf}\left(\alpha (1-u)\right)+\left[{\left(u_{c}-u\right)}^{2}+\frac{1}{2\alpha^{2}}\right]\frac{\sqrt{\pi }}{2}\mathrm{erf}\left(\alpha u\right)\\ +\frac{1}{\alpha }\left(u_{c}+\frac{u}{2}-\frac{3}{2}\right)e^{-\alpha^{2}{\left(1-u\right)}^{2}}+\frac{1}{\alpha }\left(\frac{u}{2}-u_{c}\right)e^{-\alpha^{2}u^{2}}\;\;\;\;\;\;\;\;\;\;\;\;\;\;\;\;\mathrm{for}\;\frac{1}{2}<u<1. \end{array}\right.$$

We have introduced two parameters *G*_0_ and *δχ_e_* which characterize the power spectral density of the pump light together with the parameters *β* and *τ*_e_. These four parameters are represented by the parameters *f*_h_, *γ*, *α* and *δν*_rms_, as follows. We denote the spectral half width at half maximum of the Gaussian spectrum as *f*_h_ and the sweep rate of the optical frequency as *γ*. The relations between *δχ_e_* and *f_h_* and between *β* and *γ* are given by *δ**χ_e_* = 2*πf*_h_/√ln2 and *β* = 2*πγ*, respectively and thus we have *τ*_e_ = *αδχ_e_*/*β* = *αf*_h_/(*γ*√ln2) from the definition of *α*. The power spectral density of the frequency deviation *δν(t*) is given by *H*(*f*) = *G*(2*πf*)/2*π* with *G*(*χ*) = *G*_0_exp[− (*χ*/*δχ_e_*)^2^], which is substituted into Equation (10) to obtain the mean value of *δν(t*) as *δν*_rms_^2^ = *G*_0_*δχ_e_*(√*π*)erf(*α*)/(2*π*)^2^, where the upper limit was set to *f*_u_ = *βτ*_e_/2*π*. When *α* > >1, erf(*α*) approaches unity and we have *δν*_rms_^2^ = *G*_0_*δχ_e_*√*π*/(2*π*)^2^ which is changed to *G*_0_*f*_h_√*π*/(2*π*√ln2) using the relation *δχ_e_* = 2*πf*_h_/√ln2, and thus we obtain *G*_0_ = 2*π*(√ln2)*δν*_rms_^2^/(*f*_h_√*π*). By substituting these relations into Equation (A79), we finally obtain:

(A83) $$\;\sigma_{\infty }^{\left(2\right)}=\frac{2}{\ln2}{\left(\frac{2\pi f_{h}\delta \nu_{\mathrm{rms}}}{\gamma }\right)}^{2}.$$
